# Th1 skewed immune response of whole virion inactivated SARS CoV 2 vaccine and its safety evaluation

**DOI:** 10.1016/j.isci.2021.102298

**Published:** 2021-03-10

**Authors:** Brunda Ganneru, Harsh Jogdand, Vijaya Kumar Daram, Dipankar Das, Narasimha Reddy Molugu, Sai D. Prasad, Srinivas V. Kannappa, Krishna M. Ella, Rajaram Ravikrishnan, Amit Awasthi, Jomy Jose, Panduranga Rao, Deepak Kumar, Raches Ella, Priya Abraham, Pragya D. Yadav, Gajanan N. Sapkal, Anita Shete-Aich, Gururaj Desphande, Sreelekshmy Mohandas, Atanu Basu, Nivedita Gupta, Krishna Mohan Vadrevu

**Affiliations:** 1 Bharat Biotech International Ltd, Hyderabad (BBIL), Telangana 500 078, India; 2 RCC Laboratories India Private Ltd, Hyderabad, Telangana 500 078, India; 3 Translational Health Sciences and Technology Institute (THSTI), NCR Biotech Science Cluster, PO box #04, Faridabad, Haryana 121001, India; 4 National Institute of Virology-Indian Council of Medical Research (NIV-ICMR), Pune, Maharashtra 411021, India; 5 Indian Council of Medical Research (ICMR), India, V. Ramalingaswami Bhawan, P.O. Box No. 4911, Ansari Nagar, New Delhi 110029, India

**Keywords:** Immune Response, Immunology, Virology

## Abstract

We report the development and evaluation of safety and immunogenicity of a whole virion inactivated (WVI) SARS-CoV-2 vaccine (BBV152), adjuvanted with aluminum hydroxide gel (Algel), or TLR7/8 agonist chemisorbed Algel. We used a well-characterized SARS-CoV-2 strain and an established Vero cell platform to produce large-scale GMP-grade highly purified inactivated antigen. Product development and manufacturing process were carried out in a BSL-3 facility. Immunogenicity and safety were determined at two antigen concentrations (3μg and 6μg), with two different adjuvants, in mice, rats, and rabbits. Our results show that BBV152 vaccine formulations generated significantly high antigen-binding and neutralizing antibody titers (NAb), at both concentrations, in all three species with excellent safety profiles. The inactivated vaccine formulation contains TLR7/8 agonist adjuvant-induced Th1-biased antibody responses with elevated IgG2a/IgG1 ratio and increased levels of SARS-CoV-2-specific IFN-γ^+^ CD4^+^ T lymphocyte response. Our results support further development for phase I/II clinical trials in humans.

## Highlights

• WVI SARS-CoV-2 vaccine (BBV152) was developed using genetically stable strain
• Adjuvanted vaccines (BBV152A, B, and C) induced high NAb titers in animal models
• Chemisorbed Algel with TLR7/8 agonists vaccine formulations (BBV152A & B) induced robust Th1 biased immunity
• Adjuvanted vaccines (BBV152A, B, and C) are found to be safe in animal models

## Graphical abstract

**Figure:**
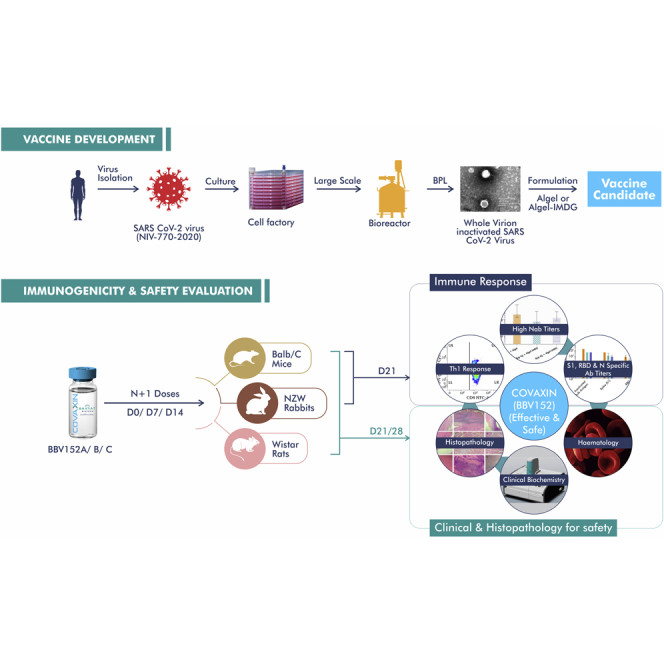

## Teaser

Immune Response ; Microbiology ; Virology

## Introduction

Severe acute respiratory syndrome coronavirus 2 (SARS-CoV-2), a novel human coronavirus, has spread across the world. SARS-CoV-2 belongs to β-genus of Sarbecovirus and is a close relative of SARS-CoV with approximately 80% sequence identity (Zhou et al., 2020). The World Health Organization (WHO) declared the disease caused by SARS-CoV-2 as coronavirus disease-19 (COVID-19), a pandemic in March 2020. So far, SARS-CoV-2 has infected more than 45 million people, causing more than 1.1 million deaths (WHO Coronavirus Disease, n.d.). It is, therefore, imperative to develop effective prophylactic and therapeutic counter measures to prevent and treat COVID-19.

Numerous vaccine candidates such as adenovirus-vectored, nucleic-acid-based, recombinant-protein-based, and inactivated vaccines are at various stages of developmental phase at either preclinical or clinical trials (Draft landscape of COVID-19 candidate vaccines, n.d.; Folegatti et al., 2020; Gao et al., 2020; Jackson et al., 2020; Keech et al., 2020; Wang et al., 2020). However, meeting the global need for billions of doses of COVID-19 vaccines will require collective effort to identify, evaluate, validate, and manufacture effective vaccines. Inactivated vaccines for viral diseases have been licensed for decades with well-established safety profiles (Sanders et al., 2015). The availability of well-characterized Vero cell manufacturing platform with proven safety have aided in rapid vaccine development of inactivated vaccines (Bhandari et al., 2014; “Global Advisory Committee (World Health Organization, 2019); Sampath et al., 2010; Singh et al., 2015; Vadrevu et al., 2020).

It has to be mentioned here that the some of the most advanced developmental stage vaccine candidates such as the inactivated vaccine (PiCoVacc) and the recombinant vaccine (CoV-RBD219N1) are aluminum adjuvant formulations. These vaccines are shown to generate high levels of NAb titers against SARS CoV 2, which could play an important role in vaccine efficacy. Hence, the development of inactivated vaccines for COVID-19 prevention appears to be a rational approach, while recognizing the fact that such inactivated vaccines with alum adjuvant specifically induce Th2-biased response. For example, inactivated SARS CoV 2 vaccine (CoronaVac, China) formulated with alum-generated Th2 response, but with low levels of Th1 response. Hence, there is no clear indication that CoronaVac induces Th1 response (Zhang et al., 2020). However, recent literature on SARS and SARS CoV 2 showed the importance of Th1-skewed immune response, in providing the protection against infection and its role in reducing the clinical severity toward subsequent infections (Janice Oh et al., 2012; Jeyanathan et al., 2020; Le Bert et al., 2020; Sekine et al., 2020). Further, antigen-specific T cell immune responses live longer than the neutralization antibodies (Ng et al., 2016) and provide long-term immunity (Cañete and Vinuesa, 2020). Hence, SARS CoV 2-specific T cell immunity is found to be critical, while developing a vaccine against SARS CoV 2. Given this, we formulated an inactivated vaccine with an adjuvant (Algel-IMDG) containing TLR7/8 agonists molecule, known to induce Th1-biased immunity (Miller et al., 2020; Shukla et al., 2012; Warshakoon et al., 2009). Here, we report the immunogenicity and safety evaluation of the whole-virion inactivated SARS-CoV-2 vaccine candidate (BBV152) formulated in Algel or Algel-IMDG, in three animal models.

## Results

### Isolation and selection of SARS-CoV-2 strain for vaccine candidate preparation

During the initial outbreak of SARS-CoV-2 in India, specimens from 12 infected patients were collected and sequenced at the Indian Council of Medical Research-National Institute of Virology (ICMR-NIV), India, a WHO Collaborating Center for Emerging Viral Infections (Sarkale et al., 2020). The SARS-CoV-2 strain (NIV-2020-770) used in developing the BBV152 vaccine candidate was retrieved from tourists who arrived in New Delhi, India (Potdar et al., 2020; Yadav et al., 2020b). The sample propagation and virus isolation were performed in the Vero CCL-81, and strain sequence was deposited in the GISAID (EPI_ISL_420545). The BBV152 vaccine candidate strain is located in the (G clade), also represented as “20A” clade that is the most prevalent strain in India (followed by “19A”) as per data represented in the next strain analysis of the Indian analysis (Hadfield et al., 2018). In terms of the overall divergence of SARS-CoV-2, this strain is 99.97% identical to the earliest strain Wuhan Hu-1 (Sardar et al., 2020). The multiple passages done in the Vero CCL-81 demonstrated the genetic stability of the virus. The next-generation sequencing (NGS) reads generated from the nucleotide sequences of the BBV152 vaccine candidate strain and its passage one at PID-3 (post-infection day 3) were found to be comparable with the SARS-CoV-2 Wuhan Hu-1 strain (Table 1). A maximum difference of 0.075% in the nucleotides was observed, indicating negligible changes in the different batches of the samples analyzed. Thus, these results showed genetic stability of the NIV-2020-770 strain, which leads to further vaccine development.

**Table 1:** Genetic stability of the BBV152 viral strain under specific passages (Vero CCL-81 passage 1 PID-3)

Reference position	Wuhan Hu-1 nucleotide	Current nucleotide	Substitution	Count of reads	Frequency of reads	Region
241	C	T	Synonymous change	10,937	99.7	5′ UTR
3037	C	T	Synonymous change	6,227	99.6	orf1ab
4809	C	T	Nonsynonymous change (S- > F)	11,561	99.91	orf1ab
14408	C	T	Nonsynonymous change (P- > L)	7,562	99.91	orf1ab
23403	A	G	Nonsynonymous change (D- > G)	13,336	99.96	S

### Vaccine candidate preparation

The seed virus was adapted to a highly characterized GMP Vero cell platform, amplified to produce the master and working virus bank. The master virus bank was well characterized based on WHO Technical Report Series guidelines (WHO-TRS_978_Annex_3.pdf, 2013) , including identity, sterility, mycoplasma, virus titration, adventitious agents, hemadsorption etc. GMP production of virus bulk was performed in the bio-safety level-3 (BSL-3) facility using bioreactors.

**Figure 1:**
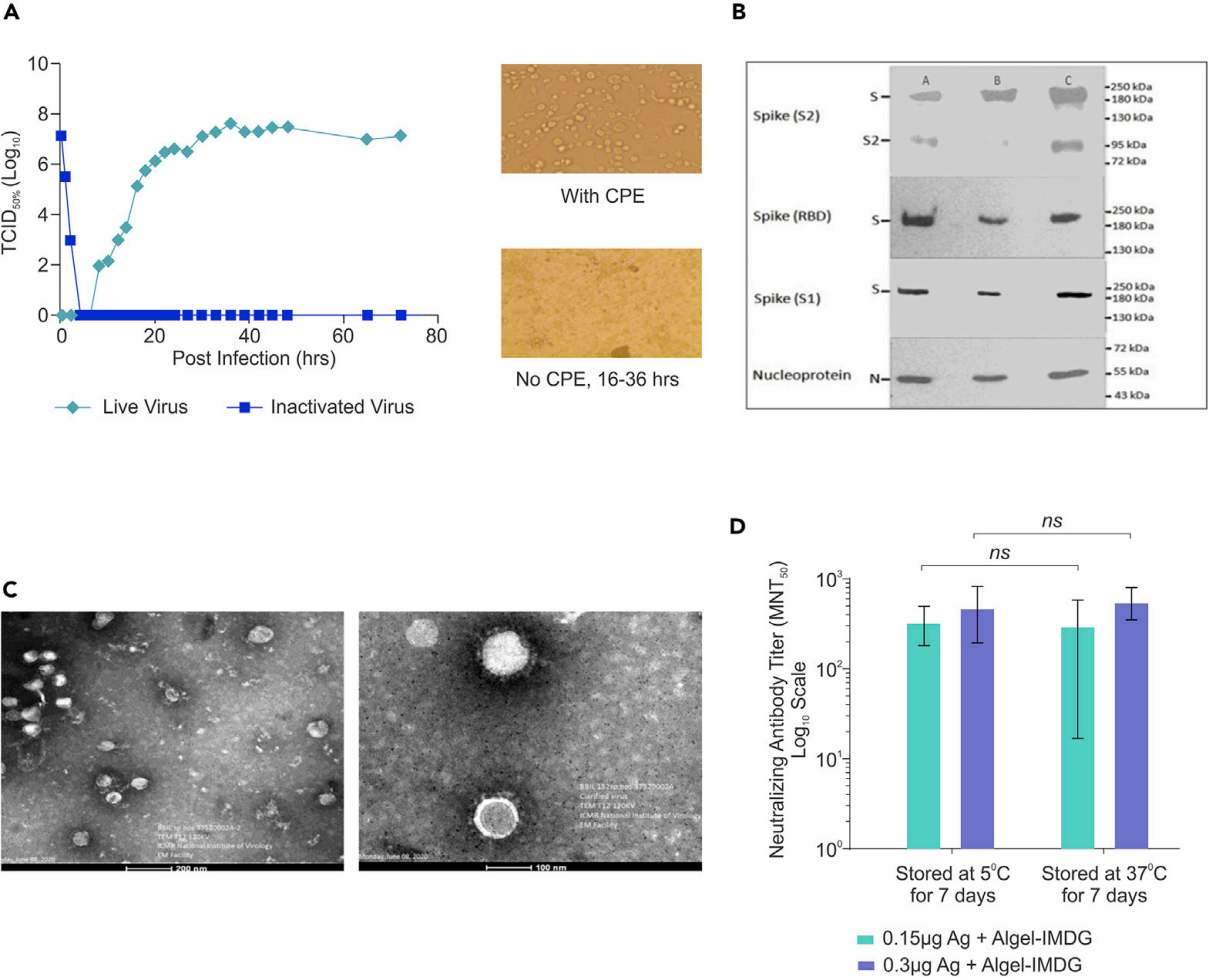
Characterization of inactivated SARS-CoV-2 and evaluation of the stability of BBV152 vaccine formulations (A) SARS-CoV-2 virus (Strain NIV-770-2020) growth kinetics and its cytopathic effect (CPE) before and after inactivation. (1) Line graph represents virus titer (10^6^–10^7^) measured by CCID_50_ at every 3 h up to 48 h and after that every 12 h (24, 27, 30, 33, 36, 39, 42), (2) microscopic images represent cells with cytopathic effect (CPE) before inactivation and no CPE after inactivation, (3) image of Vero cell monolayer with no CPE observed from 16 to 36 h; (B) Western blot analysis of purified inactivated SARS-CoV-2 produced from three production batches; the antibody used for each panel is mentioned on the left side of the image; (C) Representative electron micrograph of purified inactivated SARS-CoV-2 candidate vaccine (BBV152) at a scale bar: 100 nm (right) and 200 nm (left); (D) Bar graph represents microneutralization antibody titer of day 14 individual sera (7 days after 2^nd^ dose) collected from mice vaccinated with 1/20^th^ dose of adjuvanted formulations (0.15 μg Ag with Algel-IMDG and 0.3 μg Ag with Algel-IMDG), after subjecting them for stability at 37°C for 7 days and compared with 2–8°C. Error bars represent median with 95% CI and the statistical analysis performed using one-sample T test shown not significant.

Growth kinetics analysis revealed that the SARS-CoV-2 virus replicated to 7.0 log10 TCID_50_ between 36 and 72 h (Figure 1A). The β-propiolactone was utilized for the inactivation of the virus by mixing the virus stock at 2-8°C. Inactivation kinetics was performed with varying conditions and concentrations, and samples were collected at various time points (between 0 and 24 h, at 4-h intervals) to evaluate the cytopathic effect. Three consecutive inactivation procedures (each lasting 24 h) were performed to ensure complete viral inactivation without affecting the antigen stability (Figures 1A and 1B). Purified and inactivated whole-virion antigen produced from three production batches was also characterized by western blot for its identity using anti-Spike (S1 & S2), anti-RBD, and anti-N protein (Figure 1B) antibodies. These results demonstrated that the final purified inactivated bulk of the vaccine candidate contains spike and nucleocapsid (N) protein. The uncleaved full-length spike was detected by the spike (S2) (Figure 1B first panel), spike (RBD) (Figure 1B second panel), and spike (S1) (Figure 1B third panel) antibodies. The spike (S2) antibody also detected the S2 subunit (Figure 1B first panel). The full-length spike (S), S2 fragment (S2), and nucleocapsid (N) protein bands were of expected molecular weight (Figure 1B). Transmission electron microscopy (TEM) analysis also showed that the inactivated and purified virus particles were intact, oval-shaped, and were accompanied by a crown-like structure representing the well-defined spike protein on the virus membrane (Figure 1C), and these data corroborate with electron micrograph of the live virus (Prasad et al., 2020).

### Vaccine formulations with adjuvants and their stability

Inactivated whole-virion SARS CoV-2 (BBV152) vaccine candidates were formulated with two alum-based adjuvants: Algel (aluminum hydroxide gel) and Algel-IMDG, an imidazoquinoline class molecule (TLR7/TLR8 agonist, abbreviated as IMDG) chemisorbed onto aluminum hydroxide gel. Three vaccine formulations were prepared: first two contain 3 μg and 6 μg antigen with Algel-IMDG (BBV152A and BBV152B, respectively) and the third one contains 6μg antigen with Algel (BBV152C). To determine the stability of these formulations, Algel-IMDG vaccine formulations (BBV152A and BBV152B) were stored at 37°C and 2–8°C temperature for 7 days. These vaccine formulations were diluted 20 times (1/20^th^ human single dose (HSD), equivalent to 0.15 and 0.3 μg antigen) and administered in BALB/c mice intra-peritoneally to evaluate NAb titer by microneutralization test (MNT_50_) at 7-day post-immunization. Our results demonstrated that the both vaccine formulations are relatively stable at 37°C for 7 days, as shown by equivalent NAb titer compared with formulation stored at real time (2–8°C) temperature (Figure 1D).

### Safety evaluation of Algel-IMDG and adjuvanted vaccine formulations (BBV152)

Algel is a well-known adjuvant having been used in a large number of vaccines globally. Hence extensive safety evaluation was performed for the Algel-IMDG adjuvant alone along with three adjuvanted vaccine formulations (BBV152A, B, and C), as per the regulatory guidelines (WHO, 2013; OECD, 2020; CDSCO, 2019). Table 2 summarizes the key toxicology studies/tests performed and the observations thereof.

**Table 2:** Immunogenicity and safety studies conducted

Study type	Animal model	Test item^a^^,^^b^^,^^c^^.^^d^	Route of administration	No of animals^e^	Key test item result
Immunogenicity	BALB/c mice	PBS, antigen, adjuvanted vaccines, & adjuvants	Intraperitoneal	50 (F)	High spike (S1)-specific Ab titers and NAb titers were observed.
Immunogenicity	BALB/c mice	PBS & adjuvanted vaccines	Intraperitoneal	40 (20M + 20F)	High antigen-specific (S1, RBD, & N) Ab titers and NAb titers along with cell-mediated responses were observed.
Immunogenicity	BALB/c mice	PBS, antigen, & adjuvanted vaccines	Intramuscular	24 (12M + 12F)	Adjuvanted vaccines show high S1-specific titer when compared with antigen.
Long-term immunogenicity	BALB/c mice	PBS, adjuvanted vaccines	Intramuscular	28 (14M + 14F)	Consistent high S1-specific Ab titers & NAb titers were observed upto 98 days.
Repeated dose toxicity studies	Wistar rats	PBS, antigen, adjuvanted vaccines, & adjuvants	Intramuscular	82 (41M + 41F)	All the test items have been demonstrated to be safe from a toxicology perspective^f^.
	Swiss albino mice	PBS, adjuvanted vaccines, & adjuvants	Intramuscular	32 (16M + 16 F)	
	New Zealand white rabbits	Adjuvanted vaccines^g^	Intramuscular	10 (5M + 5F)	
Mutagenicity assay (bacterial reverse mutation)	*Salmonella typhimurium*	Algel-IMDG	–	–	
Maximum tolerated dose studies	Swiss albino mice	Algel-IMDG	Intramuscular	10 (5M + 5F)	
Maximum tolerated dose studies	Wistar rats	Algel-IMDG	Intramuscular	10 (5M + 5F)	

a Antigen: BBV152 Antigen at 3 (low), 6 (middle), & 9 μg (high).

b Adjuvanted vaccines: BBV152A, BBV152B, & BBV152C.

c Adjuvants: Algel & Algel-IMDG at 20 & 30 μg agonist.

d PBS, phosphate-buffered saline.

e M, male; F, female.

f Details are given in supplementary section.

g Preimmune sera were used as baseline titers for ELISA & PRNT_90_/MNT_50_.

Safety of Algel-IMDG was evaluated by three experiments: (1) mutagenicity assay (*in-vitro*) to determine mutagenic potential of the Algel-IMDG; (2) maximum tolerated dose test (MTD, *in-vivo*), to ensure the human intended adjuvant dose is tolerable; and (3) repeated dose toxicity study (RDT, *in-vivo*), to evaluate that repeated administration of Algel-IMDG does not cause any systemic toxicity or mortality. Mutagenicity assay performed with Algel-IMDG at various concentrations revealed that there was no substantial increase in revertant colony numbers in any of the tested strains at any dose level, in both the plate-incorporation and pre-incubation methods in the presence or absence of metabolic activation (S9 mix) (Table S1). Thus, the Algel-IMDG used in the BBV152 A and B-adjuvanted vaccine formulations was found to be non-mutagenic. Further, maximum tolerated dose study performed with single dose of Algel-IMDG also revealed that the Algel-IMDG was tolerated at the tested dose (20 μg agonist/animal) in mice and rats as demonstrated by lack of erythema, edema, or any other macroscopic lesions at the site of injection (Table S2).

Moreover, repeated administration of (N+1 dose regimen) high dose of either Algel-IMDG alone (30μg agonist/animal) in Swiss Albino mice and Wistar rats or high dose of adjuvanted vaccine formulation (9μg Ag with 30μg agonist/animal, which is more than HSD) in Wistar rats did not show any clinical illness, change in body weight (Figure S1), or histopathological changes, except inflammation at the site of injection (Figure S3) and thus established the safety of both Algel-IMDG and adjuvanted vaccine formulations at high dose.

Further, the safety of adjuvanted vaccine formulations (BBV152 A, B, and C) either at full HSD or 1/10^th^ or 1/20^th^ HSD was also evaluated to be safe in three animal models (BALB/c mice, S. albino mice, and NZW rabbits), as demonstrated by the repeated dose toxicity study with no mortality and with no changes in clinical signs, body weight gain, body temperature, or feed consumption in any of the animals. Representative data of body temperature as a parameter are shown in Table S3.

Clinical pathological parameters such as hematology, clinical biochemistry, coagulation studies, and urinalysis performed in repeated dose toxicity (RDT) studies showed that the animals administered with either adjuvanted vaccine candidates or adjuvants/antigen-alone were comparable with control (Figure S2, Table S4), except increased levels of Alpha 1- acid glycoprotein and neutrophils count on day 2 in adjuvant-alone or adjuvanted vaccine formulation groups. However, these values were comparable with control on day 21. This transient increase may be due to inflammation at the injection site after administration of the first dose. These findings were further correlated with the inflammatory reaction at the injection site observed microscopically, in the animals administered with adjuvant-alone and adjuvanted vaccine with Algel and Algel-IMDG. This inflammation was found to be slightly higher in animals that received Algel-IMDG than in animals that received Algel. However, this inflammation reduced by day 28 (Figures S3 and S4). Other than local reaction at the site of injection, no other treatment-related microscopic findings were observed in any of the animals administered with antigen or adjuvant or adjuvanted vaccine formulations. Histopathological examination of organs such as spleen, lungs, heart, and lymph nodes etc., of all animal models administered with antigen or adjuvant or adjuvanted vaccine formulations was normal (Figures S5 and S6).

### Adjuvanted vaccine formulations (BBV152) induced high neutralization antibody titers

We report immunogenicity of three BBV152 formulations in BALB/c mice and New Zealand white rabbits. BALB/c mice were administered either with full or 1/10^th^ or 1/20^th^ HSD, whereas Rabbits were immunized with intended HSD. Table 2 summarizes the immunogenicity studies performed and the observations thereof.

### Immunogenicity in BALB/c mice

**Figure 2:**
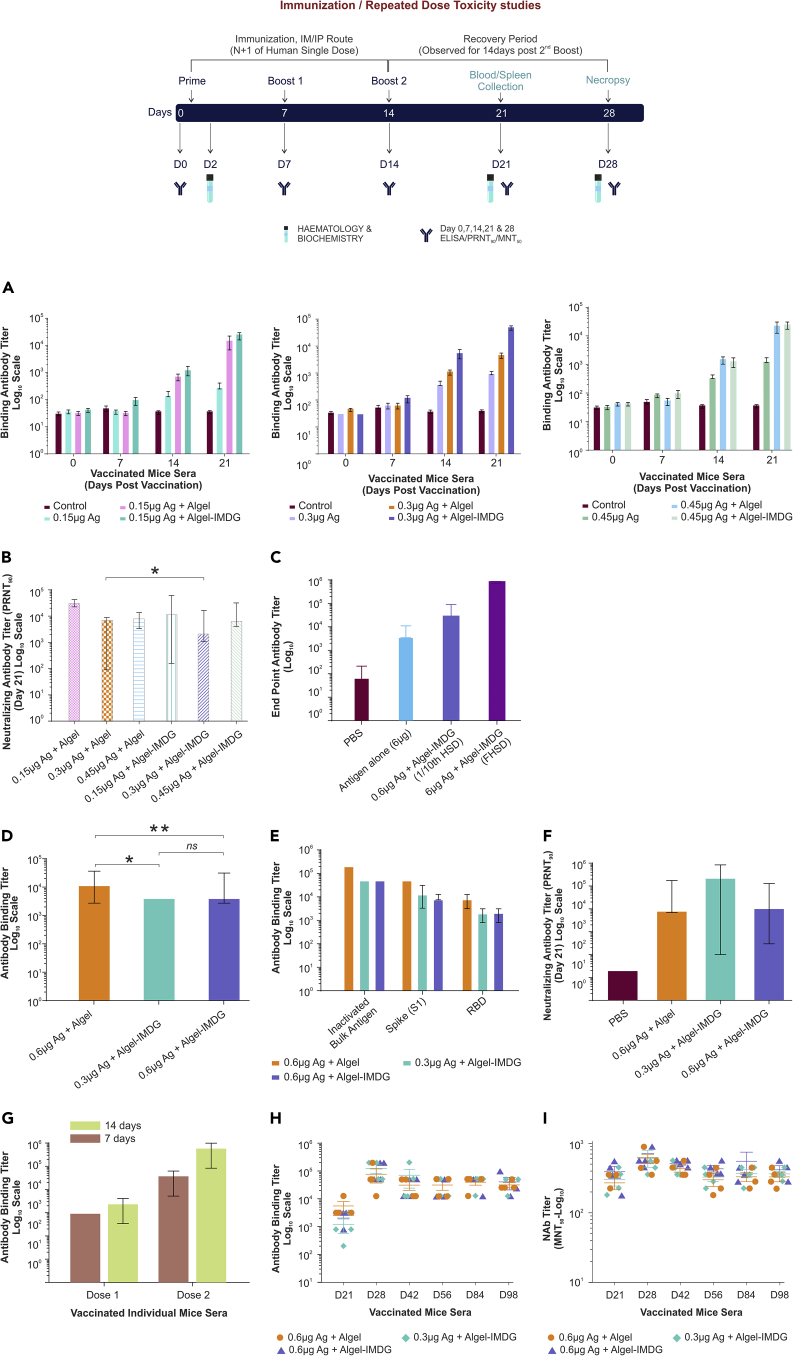
BBV152 vaccine induces high virus-specific antibody response in mice (A–I) Immune response elicited against antigens at three concentrations of antigen or adjuvanted vaccine formulations in BALB/c mice (n = 5, female) were represented. Animals were administered via IP route either with 1/20^th^ (Fig A & B) or 1/10^th^ (Fig D, E, & F) human single dose (HSD) or administered via intramuscular route with either full HSD or 1/10th dose (C, G, & H). S1-specific total IgG antibody binding titers measured using individual sera collected (A) at various time points (day 0, 7, 14, & 21) at 1/20^th^ dose; (D) on day 21 with 1/10^th^ dose; (C) on day 21 either with full HSD or 1/10^th^ dose; (G)on post-dose 1 (on day 7 or 14) and 2 (on day 14 or 28), when BALB/c mice were administered with BBV152B via IM route with different immunization schedule with an interval of 7 or 14 days, and (H) at various time points (day 21, 28, 42, 56, 84, and 98) at 1/10^th^ dose via IM route. (E) SARS-CoV-2 specific (S1, RBD, N and total inactivated antigen) antibody binding titers elicited against adjuvant vaccines (BBV152A, B & C) on day 21; neutralizing antibody titers performed by PRNT_90,_ using day 21 sera collected from BALB/c mice, when administered via IP route either with 1/20^th^ (B) or 1/10^th^ dose (F) or collected at various time points day 21, 28, 42, 56, 84, and 98 (I), when administered at 1/10^th^ dose via IM route. Antibody binding titers were performed by ELISA and neutralizing antibody titers by PRNT_90_. Bar graphs representing data represented as mean ± SD (G), mean/mean ± SEM (A, H, & I) derived from individual mice sera data analysis. For the long-term immunogenicity study, sera from four animals per group were tested for ELISA and MNT_50_ analyzed. Statistical analysis performed by (B) Wilcoxon rank test indicates significant difference between 0.3 μg antigen Algel and 0.3 μg antigen Algel-IMDG at p value < 0.05 and error bars indicate median with 95% CI, whereas in figure D, statistical analysis performed by Mann Whitney test showed significant difference at p value < 0.005 (∗∗) and <0.05 (∗), respectively. ns indicates not significant.

Initially, BALB/c mice (n = 5/group, female) were administered with adjuvanted vaccine formulations, antigen or adjuvants alone at 1/20^th^of the intended HSD (i.e., 1/20^th^ of 3 μg, 6 μg, and 9 μg/mouse or 0.15 μg, 0.3 μg, and 0.45 μg/mouse), to determine the optimal dose. ELISA titers (Figure 2A) and NAb titers (Figure 2B) determined at various time points revealed that immune response elicited against these adjuvanted vaccine formulations tested at three antigen concentrations elicited high levels of binding and NAb titer (Figures 2A and 2B). Antibody response determined on day 7 was found to be less (10^2^titer) robust or negligible compared with day 14 and day 21 with a titer of 10^3^ and 10^4^, respectively. Notably, 3 and 6 μg formulations induced high or similar spike-specific antibody titers compared with 9 μg group. Hence, adjuvanted formulation with high antigen dose (9 μg) was eliminated in further studies of the immunogenicity, whereas safety was evaluated in Wistar rats at this high dose. These results also indicated that adjuvanted vaccine formulations either with Algel or Algel-IMDG elicited high spike (S1)-specific antibody binding titers compared with antigen alone tested at all three concentrations (Figure 2A). This was further evaluated by administering BALB/c mice intramuscularly with antigen at actual HSD (6 μg Ag) either in the presence or in the absence of adjuvant (Algel-IMDG) and compared with 1/10^th^ HSD of adjuvanted vaccine formulation (0.6 μg Ag and Algel-IMDG). Immune response elicited against adjuvanted vaccine formulation (BBV152B) was significantly (1 log) higher than the antigen alone (6 μg Ag), which is comparable with 1/10^th^ HSD of adjuvanted vaccine formulation (1/10^th^ of BBV152B). These results suggest the dose-sparing effect of Algel-IMDG (Figure 2C).

Further, to assess the immunogenicity and safety of clinical batch samples, BALB/c mice (n = 10/group, 5 male and 5 female) were vaccinated via IP route with three adjuvanted vaccine formulations with Algel and Algel-IMDG at 1/10^th^ human intended single dose (0.3 and 0.6 μg/dose with Algel or Algel-IMDG). All adjuvanted vaccine formulations elicited antigen-specific binding antibodies (Figure 2D). Further, sera collected on day 21 were analyzed by ELISA to determine S1, RBD, and N-specific binding titer (Figure 2E) and showed 100% seroconversion with S1, RBD, and N protein. Analysis of plaque reduction neutralization test (PRNT_90_), performed with individual mice sera, showed high NAbs in all adjuvanted vaccines (Figure 2F).

We also compared different immunization dose schedules (day 7 versus day 14), wherein BALB/c mice were administered intramuscularly with adjuvanted vaccine (full HSD), with one group receiving second dose on day 7 and the other group on day 14 after initial immunization. Our results indicated 8-fold increase in spike-protein-specific antibody titer, when booster dose was given with 14-day interval as compared with that given on day 7 (Figure 2G).

In addition, to demonstrate long-lived immune response, BALB/c mice (n = 8/group, 4 male and 4 female) were vaccinated intramuscularly with three adjuvanted vaccine formulations (1/10^th^ HSD of BBV152A, B, and C) on day 0, 7, and 14 and evaluated antibody titer up to 12 weeks after last dose. These results revealed that the spike-specific antibodies reached peak level on day 28, and the antibody titers were sustained up to day 98, i.e., 12 weeks after last dose (Figure 2H). Similarly, we also found sustained NAb titers up to day 98 (Figure 2I), which indicates the BBV152 vaccine candidates were able to produce long-term immunity.

### Immunogenicity in New Zealand white rabbits

**Figure 3:**
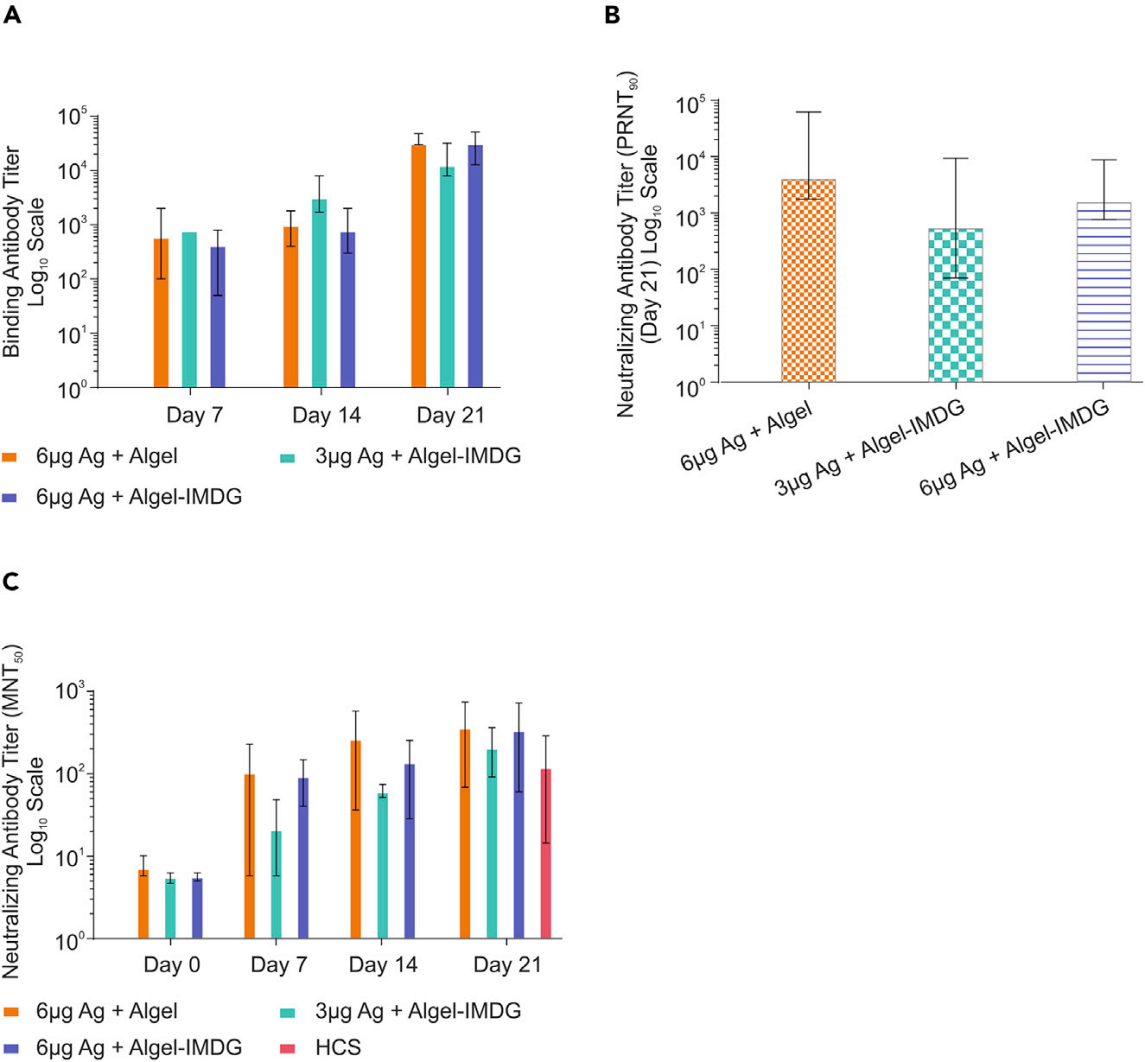
BBV152 induces robust neutralizing antibody response in rabbits New Zealand white rabbits (n = 4) were administered intramuscularly on days 0, 7, and 14 with full HSD. SARS-CoV-2-specific antibody titers were measured by ELISA. NAb tires were measured by PRNT_90_ and MNT_50_. Data Points represent median/mean of individual animal data. (A) S1-specific Ab binding titer of sera collected at various time points (day 0, 7, 14, and 21) (B) PRNT_90_ neutralizing antibody titers of day 21 sera; error bars indicate median with 95% CI, and statistical analysis performed using Wilcoxon signed rank test found no significant difference among the three adjuvanted vaccine groups. (C) MNT_50_ neutralizing antibody titers of sera collected at various time points (day 0, 7, 14, and 21) along neutralizing antibody titer (MNT_50_) with human convalescent sera (HCS) from recovered COVID-19 patients (n = 15). Samples were collected between 21 and 65 days of virological confirmation. Error bars indicate mean with 95% CI.

To assess the immunogenicity of adjuvanted vaccine formulations at full human single dose (HSD, 3 and 6 μg antigen/dose), rabbits (n = 4) were immunized intramuscularly on days 0, 7, and 14. Similar to mice, immune response in rabbits was also found to be time dependent, and not all animals were seroconverted on day 7 and showed less antibody binding titer (≥10^2^). However, on day 21, we found 100% seroconversion with spike-specific antibody binding titer of greater than or equal to 10^4^. All three formulations (BBV152A, B, and C) showed high binding antibody response (Figure 3A), with no statistically significant difference. Similarly, PRNT_90_ results showed high neutralizing antibody titers in all three adjuvanted vaccine formulations on day 21 (Figure 3B). However, there is no significant difference, and similar results were also observed by MNT_50_ titers (Figure 3C). Further, NAb titers determined by MNT_50_ were slightly higher or comparable with NAb titers of human convalescent sera collected from recovered symptomatic COVID-19 patients (Figure 3C).

### BBV152 adjuvanted with TLR7/8-adsorbed Algel-induced Th1-biased immune response

**Figure 4:**
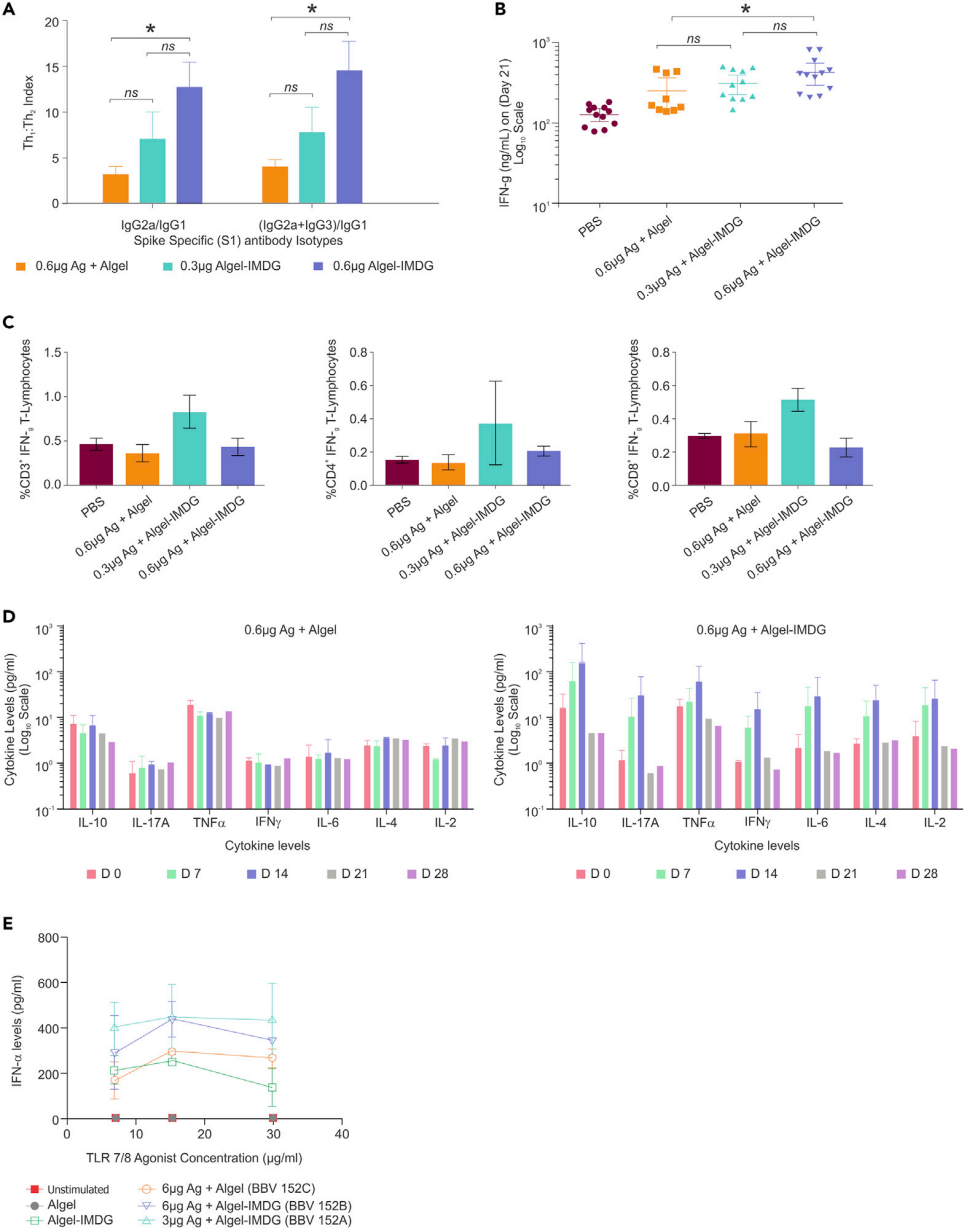
BBV152 induces a robust virus-specific T cell response Panel of figures represent cell-mediated response data generated either from vaccinated sera or from splenocytes collected from BALB/c mice or cell culture supernatant collected, after stimulation of unvaccinated huPBMCs *(ex-vivo). BALB/c mice (n = 10) were vaccinated with 1/10th HSD of adjuvanted vaccine formulations (BBV 152 A, B, and C) via the IP route.* (A*)* Th1:Th2 index generated using the formulas IgG2a/IgG1 or (IgG2a + IgG3)/IgG1, from endpoint antibody (immunoglobulin subclasses: IgG1, IgG2a, and IgG3) titer analysis measured by ELISA, using sera collected from day 21 (7 days post-third dose), from BALB/c mice, administered with 1/20th HSD of adjuvanted vaccine formulations (BBV 152 A, B, and C) via the IP route. Endpoint titer of respective immunoglobulin sub classes obtained from PBS/Algel/Algel-IMDG were taken as baseline. Error bars represent mean ± SEM. (B) Secreted IFN gamma levels estimated by ELISA, on day 21 sera (7 days post-third dose). Statistical analysis done by Mann Whitney test showed significant difference at p value < 0.05, between 0.6 μg antigen Algel and 0.6 μg Algel-IMDG. (C) Percent of CD3^+^ (Left) or CD4^+^ (Middle) or CD8^+^ (right) T lymphocytes producing IFN gamma measured by intracellular staining assay using the splenocytes of individual BALB/c mice administered with 1/10^th^ HSD. Error bars indicate mean ± SEM. (D) Cytokine profile measured on various time points using vaccinated BALB/c mice sera, when administered with adjuvanted vaccine formulations (1/20^th^ HSD via IP route). Left—BBV152C-antigen 6μg + Algel. Right—BBV152B-antigen 6μg + Algel-IMDG. Error bars indicate mean ± SDE. IFNα levels measured by ELISA from culture supernatant, when stimulated healthy PBMCs with Algel or Algel-IMDG or adjuvanted vaccine formulations (BBV152A, B, and C)). Two-fold serial dilutions of the human intended dose of adjuvanted vaccine formulations were used. Corresponding antigen or adjuvant alone concentration were also maintained simultaneously as controls. Error bars indicate mean ± SD of triplicate values.

Immunoglobulin subclasses (IgG1, IgG2a, and IgG3) were analyzed on day 14 hyperimmunized BALB/c mouse sera samples to evaluate the Th1/Th2 polarization. The average ratio of IgG2a/IgG1 or IgG2a + IgG3/IgG1 was higher in Algel-IMDG groups when compared with Algel, indicative of Th1 bias (Figure 4A). Antigen alone showed Th1-biased response at three tested different concentrations with an average Th1:Th2 index of 3; however, ELISA and PRNT_90_ titers are less compared with adjuvanted vaccine formulations. Similarly, the same sera, when measured for interferon-γ (IFNγ) by ELISA, 6-μg Algel-IMDG samples induced significantly higher responses of IFNγ compared with Algel (Figure 4B). In addition, expression of other cytokines such as interleukin (IL)-2, IL-4, IL-6, tumor necrosis factor (TNF)**-**α, IL-17A, IL-10, and interferon gamma (IFNγ) were noticeably higher in the 6-μg Algel-IMDG when compared with 6-μg Algel (Figure 4D), especially on day 7 and 14 hyperimmunized sera.

To further evaluate Th1-skewed immune response induced by Algel-IMDG, intracellular staining was performed using vaccinated mice splenocytes after stimulation with inactivated SARS-CoV-2 antigen and determined IFNγ-producing T lymphocytes. Interestingly, we found that the adjuvanted formulation BBV152A (0.3μg Ag + Algel-IMDG) showed elevated levels of IFNγ producing CD4 cell population, whereas, BBV152B formulation (0.6μg Ag + Algel-IMDG) induced comparable levels of IFNγ producing CD4^+^ T cells to the BBV152C (0.6Ag + Algel) (Figure 4C). However, there is no significant difference among the three groups. Similarly, BBV152A formulation (0.3μg Ag + Algel-IMDG) also induced higher IFNγ production in CD3^+^ and CD8^+^ T cells compared with other two formulations.

To assess the effect of adjuvants (Algel or Algel-IMDG) on immune response and understanding the critical role of IMDG in eliciting IFNα (which induces robust antibody and Th1 response), we stimulated PBMCs from healthy volunteers with adjuvants alone and adjuvanted vaccines for 36–72 h at 2-fold dilutions of BBV152A, B, and C and measured IFNα in the cell supernatant. We found that Algel-IMDG containing TLR7/8 agonists alone stimulated IFN-α but not the Algel. In addition, adjuvanted vaccine formulations with Algel-IMDG induced elevated levels of IFN-α compared with Algel formulation, demonstrating the enhanced activation of immune system by the Algel-IMDG group compared with Algel (Figure 4E).

## Discussion

Here, we report the development of a whole-virion inactivated SARS-CoV-2 vaccine candidate (BBV152A-C). The strain (NIV-2020-770), which belongs to G clade, used for this vaccine candidate is pathogenic in humans, which is the current predominant circulating strain all over the world. It is also observed that the D614G mutation increases viral neutralization (Sarkale et al., 2020). This strain also showed extensive genetic stability and appropriate growth characteristics and thus we chose NIV-2020-770 strain for further vaccine development.

Preclinical toxicity or safety evaluation of either adjuvant-alone (Algel-IMDG) or three formulations did not indicate any undesirable pathological changes and systemic toxicity, except local reactogenicity at the site of injection (Figures S3 and S4), which was attributed to the use of adjuvants in the vaccine formulation. Algel (Alum) is the most commonly used adjuvant, known to show depot formation at the site of injection, which helps the antigen for slow release (Gupta and Gupta, 2020). The microscopic findings at the site of injection in the present study showed the infiltration of macrophages and mononuclear cells indicates the activation of innate immunity. The other adjuvant namely Algel-IMDG, containing TLR7/8 agonist, induced slightly higher reactogenicity. Intra-muscular injection induces a depot effect followed by the passive trafficking of Algel particles via lymphatic flow from the interstitial space to the draining lymph nodes, as revealed by IFN-β/luciferase reporter mice (unpublished data from Dr. Sunil David, ViroVax, LLC, KS, USA). The lymph node targeting of Algel-IMDG ensures high adjuvant activity in the target organ (lymph nodes) by enabling the induction of a strong, specific, adaptive immune response while minimizing systemic exposure. Further, Algel-IMDG did not show mutagenicity in the five strains of *Salmonella typhimurium* tested. The local reaction in the studies conducted was consistent with those available in the literature for these adjuvants, which is a physiological reaction to activate immune system rather than any adverse event (Gupta and Gupta, 2020; Sellers et al., 2020).

Our results show that the vaccine formulations induced significantly elevated antigen-binding antibody and NAb responses in the immunized animals, with a distinct Th1 bias observed with Algel-IMDG adjuvanted vaccines. Although the neutralizing antibody titers are not statistically different between the antigen concentration (3 μg and 6 μg) or the nature of adjuvant, all the formulations tested have exhibited excellent immunogenicity. Recently developed, two other inactivated SARS-CoV-2 vaccine candidates (BBIBP-CorV and PiCoVacc) have been shown to induce high levels of NAb titers in mice and rats and showed protection in rhesus macaques against SARS-CoV-2 (Wang et al., 2020). Reportedly, antibodies raised against PiCoVacc also neutralized 10 representative SARS-CoV-2 strains and indicate possible broader neutralizing ability toward multiple SARS-CoV-2 strains circulating worldwide (Gao et al., 2020). Our potency results are quite favorably comparable with those reported in the literature for similar COVID-19 vaccines (Gao et al., 2020; Wang et al., 2020).

Further, in our preclinical studies, we demonstrated that all the three inactivated whole-virion SARS-CoV-2 vaccine candidates showed 100% seroconversion with high titers of antigen binding and neutralizing antibody responses. Further, the adjuvanted IMDG formulation (BBV152B) showed more than 10 times higher antibody response, compared with antigen-alone (Figure 2C), thus Algel-IMDG formulation providing dose-sparing effect. Moreover, these formulations induced immunity that is biased toward Th1-mediated response, as demonstrated by the ratio between IgG2a and IgG1 (greater than 1) (Figure 4A). In addition, secretion of anti-viral cytokines such as IL-2, IL-4, IL-6, IL-10, IL-17, TNF-alpha, and IFNγ observed on days 7 and 14 (7 days after the 1^st^ and 2^nd^ dose) (Figure 4D) and higher induction of IFN-alpha (Figure 4E) in Algel-IMDG adjuvanted formulations might have contributed to enhance activation of antigen-presenting cells, such as dendritic cells or macrophages. However, mechanism of action of Algel-IMDG in the induction of Th1-biased response is yet to be investigated. These results were further supported by our Hamster and non-human primate animal challenge study, wherein Algel-IMDG adjuvanted formulations provided early protection compared with Algel formulation, with the significant reduction in the viral load (Mohandas et al., 2021; Yadav et al., 2020a). It is also reported earlier that TLR recognition in innate cell population drives early type I IFN production, thereby promoting viral clearance and the early production of proinflammatory cytokines (Hijano et al., 2019; Stephens and Varga, 2020).

Although major research is focused on spike as the target protein for SARS CoV-2 vaccine development, there is some attention being paid toward nucleocapsid protein as a target protein, due its 90% amino acid homology and stability with fewer mutations over time (Dutta et al., 2020; Grifoni et al., 2020). Thus, it is predicted that vaccine strategies with conserved epitope regions could generate cross-protective immunity across betacoronaviruses. Le Bert et al., 2020 showed the presence of T cell responses against the structural (nucleocapsid (N) protein) and non-structural (NSP7 and NSP13 of ORF1) regions of SARS-CoV-2 in individuals convalescing from coronavirus disease 2019 (COVID-19) (Duan et al., 2020; Le Bert et al., 2020).

Further recent research findings based on bioinformatic analysis of epitope mapping revealed that nucleocapsid protein is composed of both T and B cell immunodominant epitopes (Chen et al., 2020; Sarkar et al., 2020). Earlier, animal studies conducted using DNA vaccine against SARS CoV showed that the nucleocapsid is able to produce enhanced antigen-specific humoral and cellular immune responses (Chunling et al., 2006; Zhao et al., 2005). It is also to be noted that, though, the earlier immunization studies performed in animal models against nucleocapsid protein reported to cause pneumonia (Deming et al., 2006; Yasui et al., 2008), yet, there is not much established research evidence so far on the pathogenicity of nucleoprotein in humans.

In conclusion, we believe that the ability to induce Th1-skewed immune response and the presence of conserved S and N protein in inactivated vaccine candidate formulated in Algel-IMDG would help to combat other SARS CoV-2 variants.

In our findings, we also observed high binding titers with a 100% seroconversion toward S1, RBD, and N protein. Further, high neutralization titers and protective effectiveness of COVAXIN in hamster and non-human primate models might be attributed toward the structural integrity of the inactivated whole-virion vaccine composed of target proteins, both spike and N proteins.

Bharat Biotech has developed a promising inactivated whole-virion vaccine candidate, which has now entered phase 3 clinical development (NCT04641481). The study is designed to evaluate the safety and immunogenicity of two intramuscular doses of BBV152 in healthy volunteers.

### Limitations of the study

Long-term protective efficacy of these vaccine candidates, cross-neutralization with other SARS CoV 2 variants, and mechanism of action of Algel-IMDG in inducing cell-mediated responses need to be evaluated further.

### Resource availability

#### Lead contact

Further information and requests for resources and reagents should be directed to and will be fulfilled by the lead contact, Dr. Raches Ella (ellar@bharatbiotech.com).

#### Materials availability

The materials used in this study are available upon request. This study did not generate any unique reagents.

#### Data and code availability

SARS-CoV-2 strain (NIV-2020-770) sequence was deposited in the GISAID (GenBank: EPI_ISL_420545). Primary data will be available upon request. This study did not generate any new software code.

## Methods

All methods can be found in the accompanying transparent methods supplemental file.

## Supplemental information

Document S1. Transparent methods, Figures S1–S6, and Tables S1–S4
