# Supplementary material for: Th1 skewed immune response of whole virion inactivated SARS CoV 2 vaccine and its safety evaluation
Source: iScience. 2021 Mar 10;24(4):102298. doi: 10.1016/j.isci.2021.102298 (PMC7944858; doi:10.1016/j.isci.2021.102298)
Supplement: Document S1. Transparent methods, Figures S1–S6, and Tables S1–S4 [file mmc1.pdf]

iScience, Volume *24*

Supplemental information

Th1 skewed immune response of whole virion inactivated SARS CoV 2 vaccine and its safety evaluation

Brunda Ganneru, Harsh Jogdand, Vijaya Kumar Daram, Dipankar Das, Narasimha Reddy Molugu, Sai D. Prasad, Srinivas V. Kannappa, Krishna M. Ella, Rajaram Ravikrishnan, Amit Awasthi, Jomy Jose, Panduranga Rao, Deepak Kumar, Raches Ella, Priya Abraham, Pragya D. Yadav, Gajanan N. Sapkal, Anita Shete-Aich, Gururaj Desphande, Sreelekshmy Mohandas, Atanu Basu, Nivedita Gupta, and Krishna Mohan Vadrevu

Supplemental Information

Supplementary figures and legends

Figure S1: Percent Body Weight Gain in Wistar Rats.

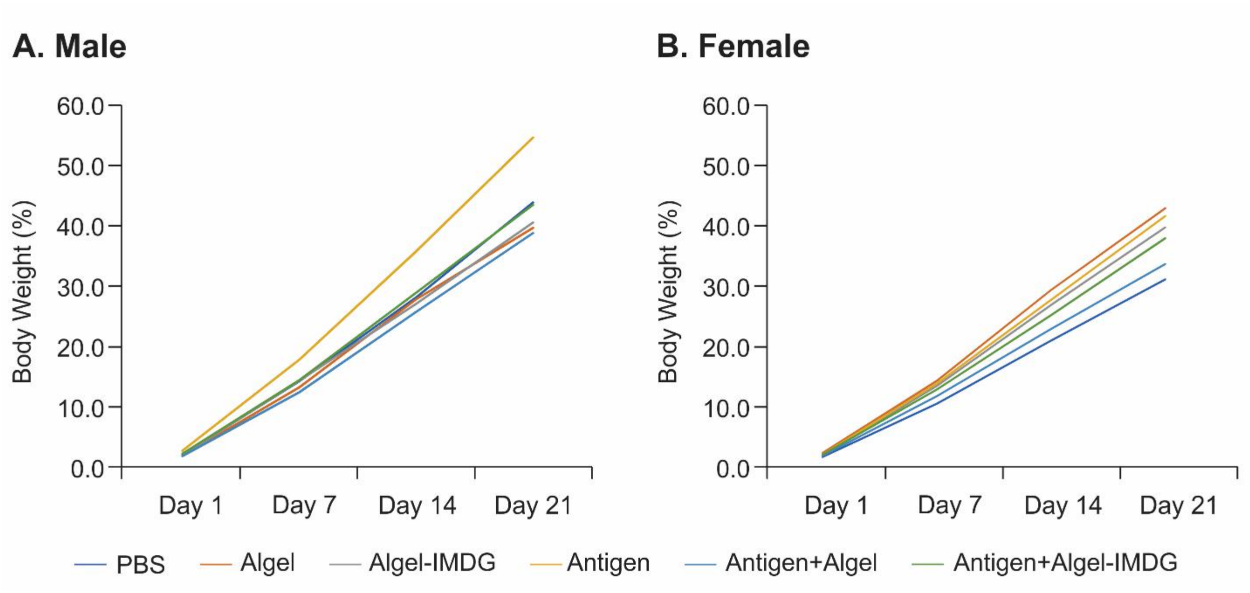

Figure S1:*Percent body weight gain recordedin (A) Male; (B) Female Rats during the experimental period (Day 1, 7, 14, and 21), when administered withN+1 dose high dose of either antigen (9 μg) or Algel or Algel-IMDG (30 μg agonist) or adjuvanted vaccines (Antigen+Algel or Antigen+Algel-IMDG). PBS denotes Phosphate buffered saline. Line graph represents Mean percent body weight gains. Related to Table 2*

Figure S2: Safety evaluation of Differential leucocyte counts in vaccinated Wistar Rats.

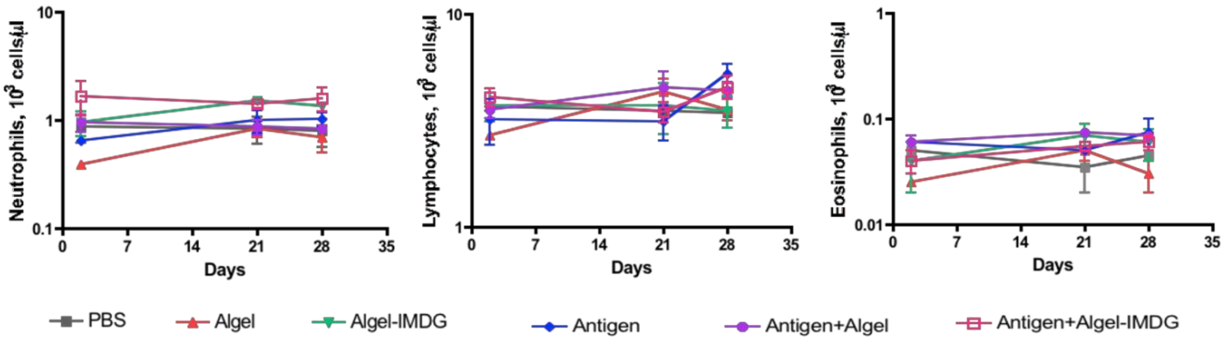

Figure S2: *Representative haematology parameters such as neutrophils, lymphocytes, eosinophils measured on day 2, 21, and 28 were shown from the Wistar rats administered with phosphate buffer saline (PBS) or antigen (9µg) or Algel or Algel-IMDG (30 µg agonist) alone and adjuvanted vaccine formulations with Algel or Algel-IMDG (antigen+ Algel or Algel-IMDG (30µg agonist). Error bars signify Means ± S.D. Related to Table 2*

Figure S3: Algel-IMDG induces more inflammatory reaction at the site of injection (quadriceps muscles).

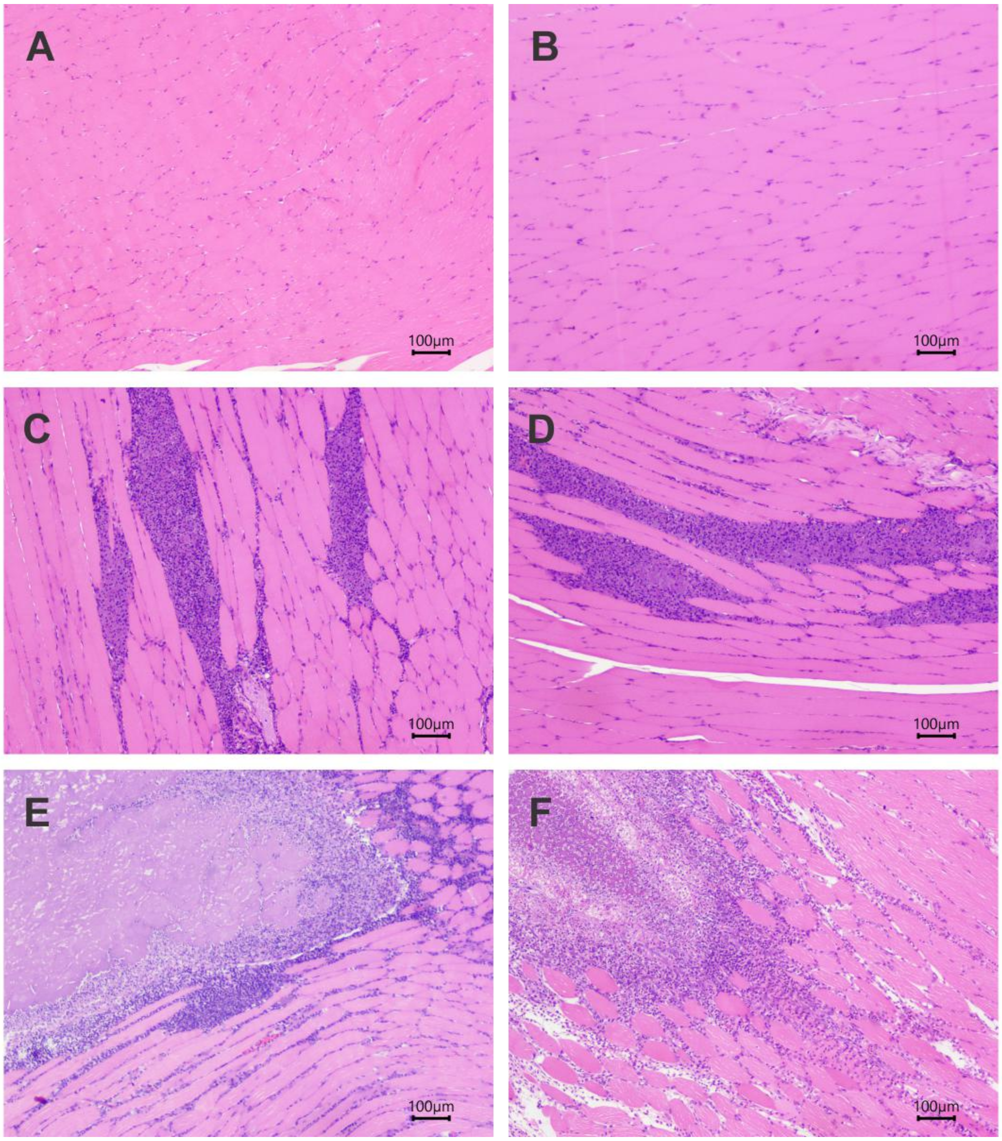

Figure S3: *Representative photomicrographs of the site of injection (quadriceps muscles) from Swiss Albino mice (Left Column) and Wistar rats (Right Column) showing macrophage infiltration containing bluish stained material in Algel (C and D) and Algel-IMDG showing chronic inflammation around test item deposits (E & F, 30 µg agonist). Injection site from PBS group(A and B) used as control for comparison. PBS denotes Phosphate buffered saline. Site of injection tissue stained with hematoxylin and eosin at magnification 4X. Related to Table 2*

Figure S4: Reduction of Macrophage Infiltration at The Site of Injection during the recovery phase

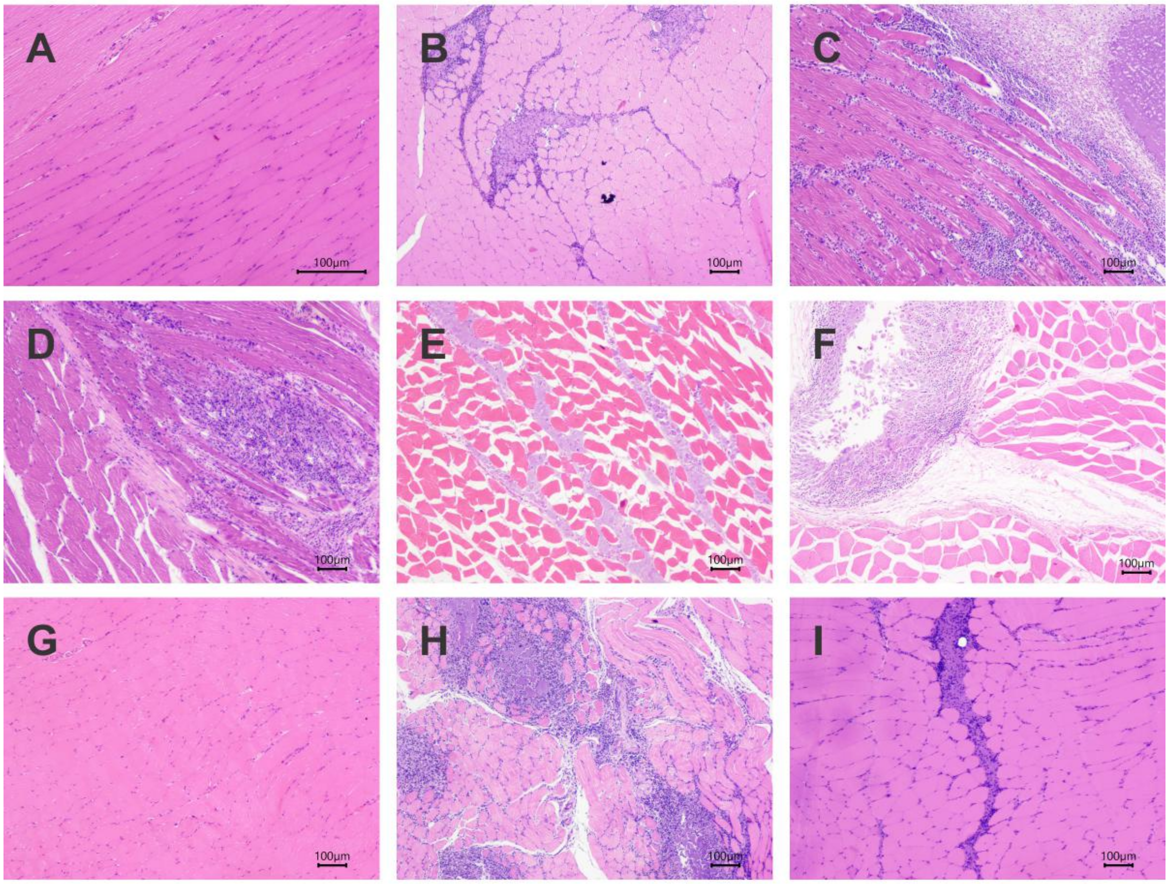

Figure S4: ***Representative photomicrographs of the site of injection (quadriceps muscles) from Wistar rats ().*** *Animals vaccinated with (A) Phosphate buffer Saline, (B) Antigen (9μg) + Algel and (C) Antigen (9μg) + Algel-IMDG (30 μg agonist). B&C shows inflammation around the site of injection, indicates infiltration of inflammatory cells and (D) Recovery group i.e., Antigen (9μg) + Algel-IMDG (30 μg agonist) showing the reduction in theinflammatory reaction.* ***New Zealand White Rabbits:*** *Animals administered with Full HSD (E) Antigen (6 μg) + Algel, (F) Antigen (6 μg) + Algel-IMDG (15μg agonist) showed macrophage infiltration (hematoxylin and eosin, original magnification was 4X).****Swiss Albino mice.*** *Animals vaccinated with Full HSD (G) Phosphate buffer Saline, (H) Antigen (6 μg) + Algel-IMDG (15μg agonist) group showing chronic inflammation around test item deposits, (I) Recovery group Antigen (6 μg) + Algel-IMDG (15μg agonist) showing thereduction in the inflammatory reaction. (hematoxylin and eosin, original magnification was 4X). Related to Table 2*

Figure S5: Photomicrographs of tissues of Wistar Rats

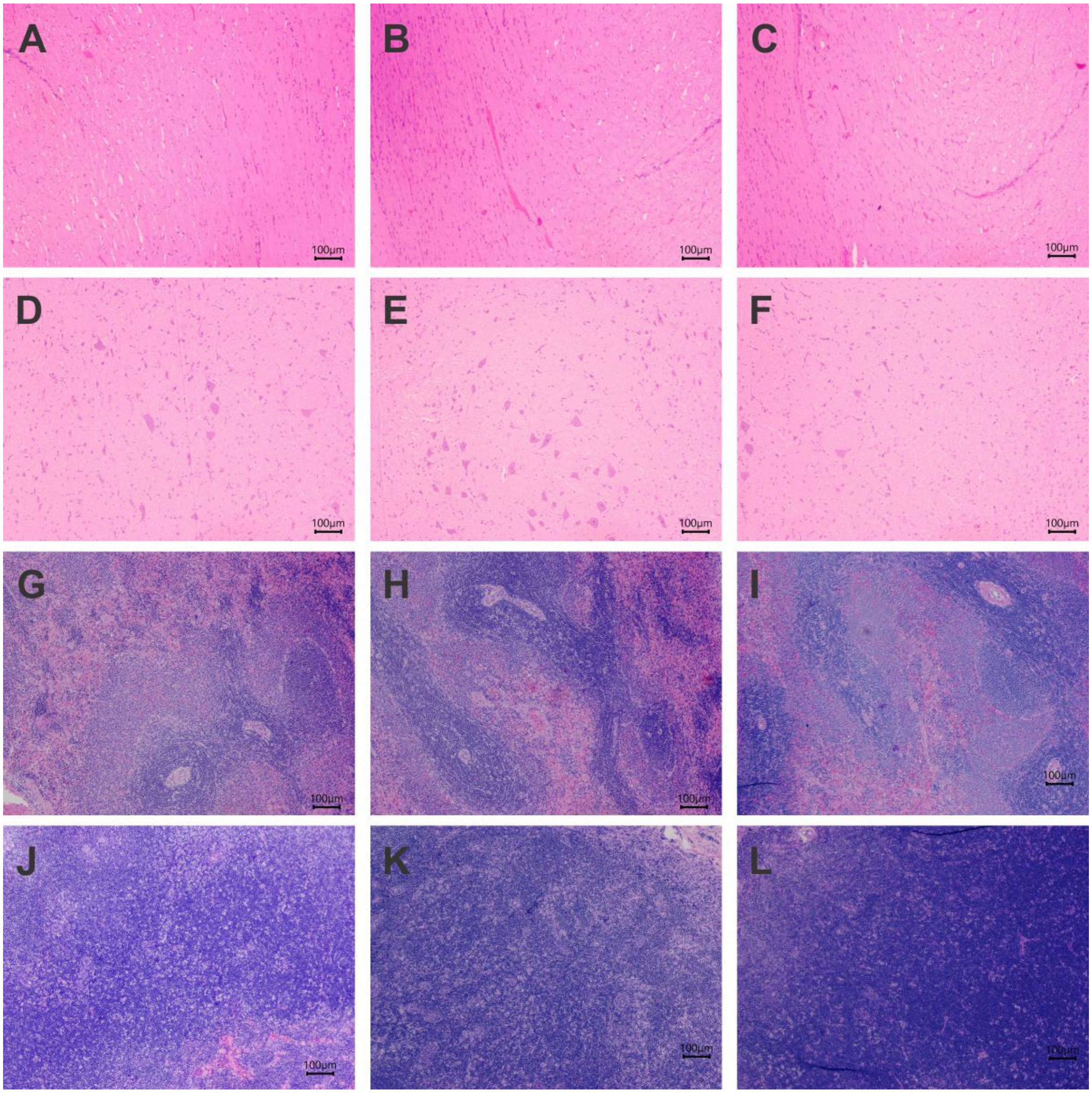

Figure S5: *Representative photomicrographs of Heart (A,B & C), Brain (D, E & F), Spleen (G, H & I) and inguinal lymph nodes (J, K & L) from Wistar Rats, when administered with PBS (control, Left Column) or high dose of adjuvanted vaccine formulations (Antigen 9μg + Algel, Middle Column) or Algel-IMDGat 30μg agonist (Right Column).These organs were within normal histological limits, when stained with hematoxylin and eosin shown under 4X original magnification. Related to Table 2*

Figure S6: Representative photomicrographs of Liver, Kidney, and Lungs from New Zealand White Rabbits.

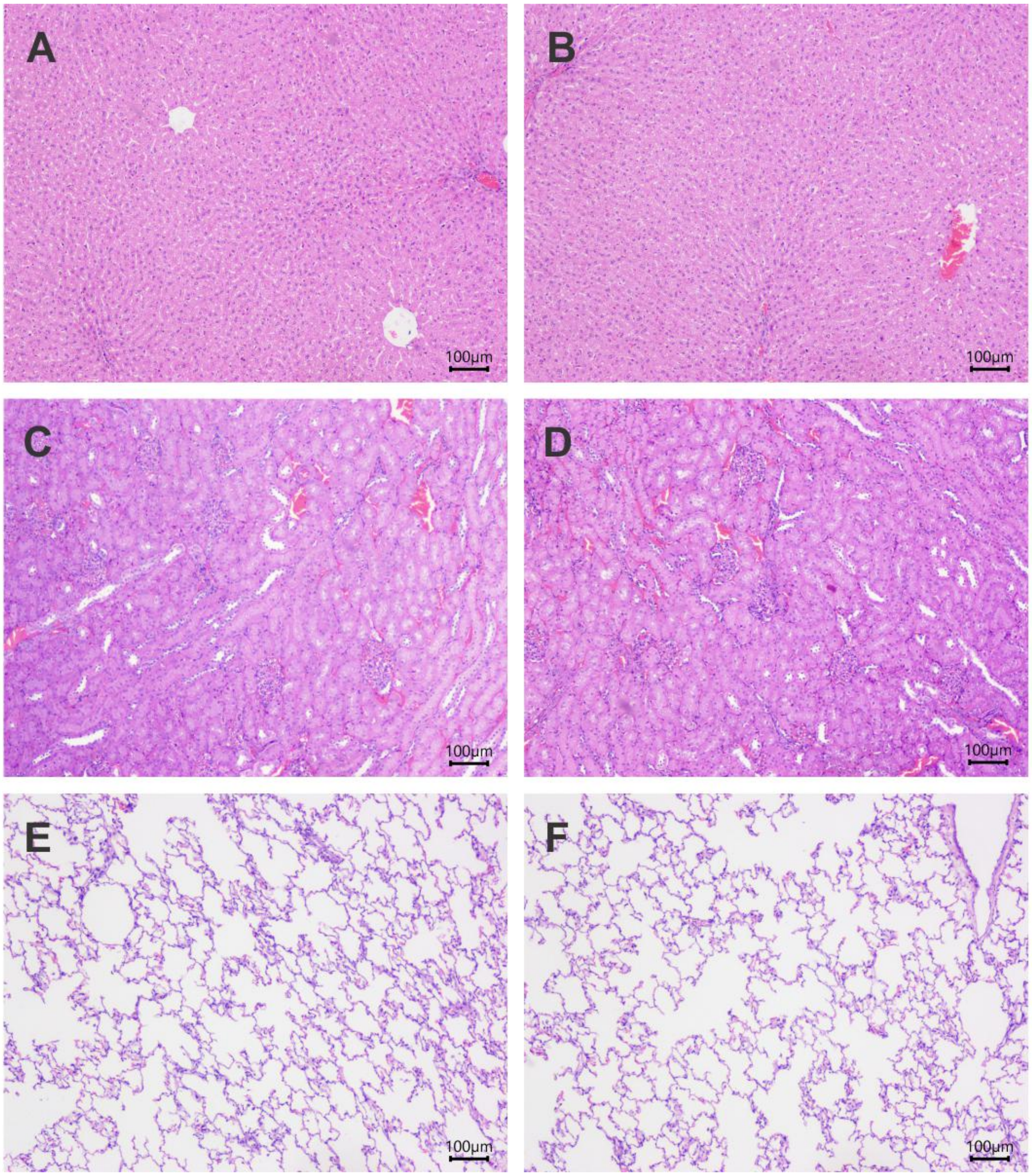

Figure S6: *Representative photomicrographs of Liver (A & B), Kidneys (C & D) and Lungs (E & F) from NZW rabbits, when administered with adjuvanted vaccines,6µg Ag + Algel (Left Column) and 6µg Ag + Algel-IMDG (Right Column) at Full HSD. Histopathological sections of all organs were within normal limits.when stained with hematoxylin and eosin shown under 4X original magnification. Related to Table 2*

Supplementary Tables

Table S1: Mutagenicity Assay

Algel-IMDG (agonist Concentration (µg/plate)	Plate Incorporation Method					Pre-Incubation Method				
	**TA1537**	**TA1535**	**TA98**	**TA100**	**TA102**	**TA1537**	**TA1535**	**TA98**	**TA100**	**TA102**
	**In the Presence of Metabolic Activation (+S9) (No. of Revertant Colony count/plate)**					**In the Presence of Metabolic Activation (+S9) (No. of Revertant Colony count/plate)**				
0.00	5.00±1.41	13.50±0.71	22.00±2.83	122.00±5.66	259.00±15.56	5.50±0.71	13.00±1.41	23.00±2.83	124.00±5.66	257.00±9.90
0.3	7.50±0.71	16.00±1.41	26.50±2.12	123.00±4.24	265.00±9.90	8.00±0.00	16.00±2.83	26.50±2.12	130.00±8.49	266.00±5.66
0.95	6.00±1.41	14.00±2.83	24.00±2.83	122.00±2.83	261.00±9.90	6.00±1.41	14.50±2.12	23.50±3.54	126.50±4.95	259.00±4.24
3.0	6.50±0.71	14.50±0.71	26.00±1.41	123.50±3.54	263.00±9.90	6.50±0.71	15.00±2.83	24.50±2.12	128.00±8.49	264.00±8.49
9.5	5.50±0.71	15.00±2.83	23.50±2.12	121.50±4.95	260.00±8.49	7.50±0.71	14.00±2.83	25.50±2.12	125.50±6.36	261.00±9.90
30	7.00±1.41	15.50±2.12	25.50±2.12	122.50±4.95	262.00±8.49	7.00±1.41	15.50±2.12	25.00±2.83	127.50±6.36	263.00±9.90
**PC**	178.00±19.80	408.00±22.63	1032.0±56.57	1428.00±118.79	1542.00±161.22	134.00±8.49	354.00±25.46	1116.00±50.91	1584.00±101.82	1722.00±93.34
	**Absence of Metabolic Activation (-S9)**					**Absence of Metabolic Activation (-S9)**				
0.00	4.50±0.71	12.00±2.83	21.00±4.24	119.00±1.41	255.00±18.38	4.50±0.71	12.50±2.12	21.50±2.12	122.00±5.66	253.00±15.56
0.3	7.00±1.41	15.00±1.41	25.00±2.83	120.50±3.54	261.00±12.73	7.00±1.41	15.50±2.12	25.00±2.83	127.50±6.36	261.00±9.90
0.95	5.50±0.71	13.00±2.83	23.50±2.12	119.50±4.95	257.00±12.73	5.50±0.71	13.00±2.83	23.00±2.83	123.50±4.95	255.00±9.90
3.0	6.50±0.71	14.50±2.12	24.50±2.12	121.00±7.07	259.00±12.73	6.00±0.00	14.50±2.12	24.50±0.71	126.00±5.66	259.50±6.36
9.5	5.00±0.00	13.50±2.12	23.00±2.83	118.00±4.24	256.00±11.31	5.00±1.41	13.50±2.12	23.50±2.12	124.00±5.66	257.00±7.07
30	6.00±1.41	14.00±2.83	24.00±1.41	120.00±5.66	260.00±8.49	6.50±0.71	15.00±2.83	24.00±2.83	125.00±5.66	258.00±11.31
**PC**	184.00±16.97	1066.00±189.50	820.00±39.60	1398.00±76.37	1746.00±195.16	184.00±16.97	996.00±175.36	716.00±73.54	1314.00±110.31	1818.00±59.40

PC: Positive Control; Values represented are in Mean±SD; TA1537, TA1535, TA98, TA100 and TA102 are five *Salmonella typhimurium* strains used to perform mutagenicity assay.
Related to Table 2

Table S2: Skin Reactions observed in Maximum Tolerated Dose Study in Wistar Rats

**Animal Nos.**	**Sex**	**Erythema (Hours)**			**Edema (Hours)**		
		**24**	**48**	**72**	**24**	**48**	**72**
01	Male	0	0	0	0	0	0
02	Male	0	0	0	0	0	0
03	Male	0	0	0	0	0	0
04	Male	0	0	0	0	0	0
05	Male	0	0	0	0	0	0
06	Female	0	0	0	0	0	0
07	Female	0	0	0	0	0	0
08	Female	0	0	0	0	0	0
09	Female	0	0	0	0	0	0
10	Female	0	0	0	0	0	0

Related to Table 2

Table S3: Summary of Body Temperature recorded in Repeated Dose Toxicity study in NZW Rabbits, after the administration of Adjuvanted vaccine

Group	Sex	Body Temperature (°C)									
		Day0 (Dose-1)			Day 7 (Dose-2)			Day14 (Dose-3)			Day21
		P.T	3 Hour	24 Hour	P.T	3 Hour	24 Hour	P.T	3 Hour	24 Hour	
6µgAg+ Algel	M	38.4 ± 0.07	38.4 ± 0.14	38.4 ± 0.07	38.3 ± 0.00	38.4 ± 0.07	38.5 ± 0.07	38.5 ± 0.00	38.7 ± 0.07	38.6 ± 0.07	38.6 ± 0.07
	F	38.4 ± 0.07	38.5 ± 0.07	38.4 ± 0.14	38.4 ± 0.21	38.6 ± 0.07	38.5 ± 0.07	38.6 ± 0.07	38.7 ± 0.07	38.6 ± 0.07	38.7 ± 0.07
6µgAg+ Algel-IMDG	M	38.3 ± 0.14	38.4 ± 0.14	38.4 ± 0.14	38.4 ± 0.21	38.4 ± 0.00	38.5 ± 0.00	38.4 ± 0.07	38.6 ± 0.07	38.5 ± 0.07	38.6 ± 0.07
	F	38.4 ± 0.14	38.5 ± 0.07	38.5 ± 0.07	38.3 ± 0.14	38.5 ± 0.00	38.5 ± 0.14	38.3 ± 0.14	38.6 ± 0.07	38.6 ± 0.07	38.6 ± 0.07

Key: °C = Degree Centigrade; A. No. = Animal Number; P.T = Pre-treatment; Values represented in Mean ± SD. *Related to Table 2*

Table S4: Summary of Clinical Biochemistry parameters observed in NZW Rabbits

GROUP		GLU	UREA	CREA	CHOL	TRIGL	AST	ALT	ALP	BIL	Na	K	Cl	TPO	ALB	GLB	A/G
		mmol/L	mmol/L	µmol/L	mmol/L	mmol/L	U/L	U/L	U/L	µmol/L	mmol/L	mmol/L	mmol/L	g/L	g/L	g/L	–
**Day 0**																	
6µgAg+ Algel (*n=4*)	M	8.43 ±1.22	9.40 ±0.04	89.4 ±15.20	1.15 ±0.01	1.39 ±0.27	41.5 ±16.69	50.25 ±11.53	166.3 ±24.25	0.4 ±0.28	146.3 ±2.97	4.72 ±0.73	101.4 ±4.53	63.4 ±1.48	56.96 ±4.89	6.4 ±3.39	10.6 ±6.36
	F	7.99 ±0.24	11.39 ±1.26	92.9 ±12.37	1.53 ±0.64	0.96 ±0.06	45.4 ±28.28	79.15 ±32.74	114.6 ±10.61	0.4 ±0.28	147.9 ±2.76	5.00 ±0.38	102.1 ±1.20	63.2 ±5.16	56.31 ±0.55	6.8 ±5.66	12.9 ±10.82
6µgAg+ Algel-IMDG (*n=4*)	M	9.05 ±0.99	13.74 ±2.33	109.0 ±8.34	1.20 ±0.95	0.92 ±0.11	36.9 ±16.40	86.20 ±23.19	110.2 ±73.82	0.2 ±0.00	148.3 ±0.21	6.13 ±0.45	102.8 ±3.11	74.7 ±11.03	64.59 ±8.05	10.1 ±2.97	6.6 ±1.13
	F	9.80 ±0.54	7.64 ±0.82	84.7 ±15.20	1.53 ±0.50	0.63 ±0.31	32.8 ±15.41	52.15 ±10.82	178.1 ±53.81	0.2 ±0.00	147.3 ±2.05	5.26 ±0.76	103.3 ±2.19	66.2 ±8.70	58.89 ±13.82	7.3 ±5.09	11.6 ±9.97
**Day 2**																	
6µgAg+ Algel (*n=4*)	M	7.08 ±0.311	9.77 ±0.47	79.2 ±17.54	1.21 ±0.21	0.97 ±0.66	19.8 ±1.27	52.30 ±15.98	123.7 ±31.40	0.6 ±0.57	137.1 ±3.39	4.18 ±0.34	98.5 ±2.12	59.5 ±2.12	53.64 ±6.06	5.9 ±4.03	12.4 ±9.48
	F	8.33 ±0.523	12.60 ±0.50	101.5 ±12.94	2.03 ±0.58	1.21 ±0.75	25.2 ±0.78	68.65 ±15.34	92.7 ±1.77	0.2 ±0.00	141.3 ±0.07	3.99 ±0.23	101.0 ±1.34	59.7 ±5.09	53.68 ±0.16	6.0 ±4.95	13.6 ±11.17
6µgAg+ Algel-IMDG (*n=4*)	M	8.84 ±0.03	10.48 ±3.81	111. ±27.58	0.97 ±0.81	0.85 ±0.02	29.4 ±9.40	67.25 ±17.89	97.5 ±71.98	0.4 ±0.28	141.8 ±0.92	4.37 ±0.14	100.7 ±3.89	65.3 ±8.06	55.40 ±5.95	9.9 ±2.12	5.7 ±0.64
	F	8.28 ±0.04	6.20 ±0.62	70.9 ±25.60	1.90 ±0.05	0.74 ±0.09	27.9 ±11.17	45.6 ±7.00	145.8 ±12.73	0.4 ±0.28	142.4 ±0.71	5.01 ±0.22	105.5 ±2.40	60.9 ±4.67	52.74 ±7.26	8.2 ±2.55	7.0 ±3.04
**Day 23**																	
6µgAg+ Algel (*n=4*)	M	5.34 ±0.53	7.77 ±0.59	80.0 ±4.03	0.83 ±0.13	0.68 ±0.07	19.2 ±2.47	38.40 ±13.72	110.4 ±36.35	0.0 ±0.28	140.6 ±0.00	3.75 ±0.91	97.5 ±3.04	56.2 ±1.91	51.54 ±1.61	4.6 ±0.28	11.2 ±0.28
	F	6.42 ±0.40	8.33 ±0.60	78.8 ±16.48	1.35 ±0.38	0.69 ±0.03	23.6 ±3.89	52.75 ±12.80	120.5 ±34.01	0.0 ±0.28	139.8 ±0.92	4.53 ±1.33	99.3 ±0.85	53.7 ±7.35	49.74 ±4.93	4.0 ±2.40	15.0 ±7.85
6µgAg+ Algel-IMDG (*n=4*)	M	5.31 ±0.74	9.22 ±0.06	96.5 ±10.68	0.74 ±0.33	0.86 ±0.25	49.0 ±6.43	81.35 ±7.57	100.4 ±85.21	-0.6 ±1.98	141.2 ±5.16	5.82 ±1.62	98.8 ±1.13	62.4 ±2.26	53.33 ±3.78	9.1 ±6.01	7.7 ±5.52
	F	6.37 ±0.93	7.25 ±0.26	77.2 ±2.69	1.33 ±0.47	0.54 ±0.05	30.5 ±10.54	34.3 ±8.91	117.8 ±39.74	-2.8 ±3.32	141.2 ±3.04	5.54 ±3.22	103.8 ±0.21	57.8 ±0.14	50.54 ±0.19	7.3 ±0.28	7.0 ±0.35

Abbreviations: GLU- Glucose, CREA- Creatinine, CHOL- Cholesterol, total, TRIGL- Triglycerides ,AST- Aspartate aminotransferase ,ALT-Alanine aminotransferase, ALP- Alkaline phosphatase, BIL- Bilirubin, Na- Sodium, K- Potassium, Cl- Chloride, TPO- Protein, total, ALB -Albumin, GLB- Globulin, A/G - Albumin-globulin ratio. *Related to Table 2*

Transparent Methods

Key Resources Table

REAGENT or RESOURCE	SOURCE	IDENTIFIER
**Antibodies**		
Anti- S1 protein rabbit polyclonal Antibody	Ella Foundation, Hyderabad, India	N/A
Rabbit polyclonal Anti-SARS-CoV-2 spike (S2) protein	Abgenex Private Limited, Bhubaneshwar, Odissa, India	Cat# 11-2002
Anti-Spike (RBD) protein rabbit polyclonal Ab	Ella Foundation, Hyderabad, India	N/A
Rabbit polyclonalAnti-SARS-CoV-2 Nucleocapsid protein	Abgenex Private Limited, Bhubaneshwar, Odissa, India	Cat# 11-2003
Anti-rabbit IgG whole molecule peroxidase	Sigma-Aldrich, USA	Cat# A6154
HRP-labeled goat anti-human IgG (gamma chain)	Invitrogen, USA	Cat# 62-8420
Goat Anti-mouse IgG HRP Conjugate	Sigma-Aldrich, USA	Cat#4416
Goat Anti-Rabbit IgG HRP Conjugate	R&D Systems, Thermo Fisher Scientific	Cat#HAF008; 65-6120
Immunoglobulin subclass Goat anti-mouse (IgG1, IgG2a & IgG3) – HRP Conjugate	Santa Cruz Biotechnology, USA	Sc-2060, Sc-2061, Sc-2972
APC-Cy™7 Rat Anti-Mouse CD3, clone: 17A2,	BD biosciences, CA, USA	Cat # 560590
FITC Rat Anti-Mouse CD4 (Clone: H129.19,	BD biosciences, CA, USA	Cat # 553650
PE-Cy™7 Rat Anti-Mouse CD8a (Clone: 53-6.7,	BD biosciences, CA, USA	Cat # 552877
BV421 Rat Anti-Mouse IFN-γ, Clone: XMG1.2	BD biosciences, CA, USA	cat # 560660
**Bacterial and Virus Strains**		
SARS CoV-2	National Institute of Virology (WHO Collaborating Center for Emerging Viral Infections), Pune, India	NIV-2020-770, Global Initiative on Sharing All Influenza Data (GISAID): EPI_ISL_420545
Salmonella typhimurium strains TA 1535, TA 1537, TA 100, TA 98, TA 102	Moltox, USA	LO No#: 5241D (PART No: 71-1535L); LO No#: 5246D, PART No: 71-1537L; LO No#: 5251D, PART No: 71-100L; 5317D, PART No: 71-1598L; 5260D, PART No: 71-102L

**Biological Samples**		
Bovine Serum Albumin	Sigma-Aldrich	Cat#A3059
Human Convalescent Sera	Gandhi Medical Hospital, Hyderabad, Telangana.	N/A
**Chemical, Peptides and Recombinant Proteins**		
β-propiolactone	Ferak	N/A
Aluminium hydroxide (Algel)	CRODA, Denmark	CAS No. 21645-51-2 Batch No. 0001606459
Imidazoquinoline class molecule (TLR7/8 agonist)	ViroVax LLC, KS, USA	N/A
Algel-IMDG	Bharat Biotech International Limited	N/A
Phorbolmyristate acetate (PMA)	Sigma-Aldrich, USA	Cat # P8139
Ionomycin	Sigma-Aldrich, USA	Cat # I0634
Golgistop (monensin)	BD biosciences, CA, USA	Cat # 554724
SARS CoV2 Spike 1 (S1) protein-His tag	Syngene, Bangalore, India	Batch No. PRB026913
SPIKE RBD PROTEIN, HIS TAG (ACTIVE)	GeneTex, California, USA	Cat# GTX01546-PRO
SPIKE S1 PROTEIN, HIS & AVL TAG (ACTIVE)	GeneTex, California, USA	Cat# GTX01548-PRO
NUCLEOCAPSID PROTEIN	GeneTex, California, USA	Cat# GTX135592-PRO
3,3′,5,5′-Tetramethylbenzidine (TMB)	DenovaBiolabs, Bangalore, India	Cat# AR1002
**Critical Commercial Assays**		
Cytokine Bead Array Kit	BD biosciences, CA, USA	Cat. # 560485
Cytoperm/permeabilization kit	BD biosciences, CA, USA	Cat. # 554722
Protein transport inhibitor	BD biosciences, CA, USA	cat # 554724
QIAamp Viral RNA mini kit	QIAGEN, Germany	Cat No.ID: 52904
VeriKine Human Interferon Alpha ELISA Kit	PBL Assay Science, USA	Cat log# 41100
Mouse IFNγ ELISA Kit	R&D systems, Minnesota, USA	Cat# DY485-05
Rat Alpha-1-AGP	Life Diagnostics, USA	Cat# AGP-2
Rabbit C-Reactive Protein	Life Diagnostics, USA	Cat# CRP-10
Mouse Alpha-1-AGP	Life Diagnostics, USA	Cat# AGP-1
**Deposited Data**		
SARS CoV-2 Strain	National Institute of Virology (WHO Collaborating Center for Emerging Viral Infections), Pune, India	Strain No# NIV-2020-770, Global Initiative on Sharing All Influenza Data (GISAID): EPI_ISL_420545
**Experimental Models: Cell Lines**		
Vero Cell Lines	ATCC, Manassas, USA	Cat. # Vero ATCC CCL-81
**Experimental Models: Organisms/Strains**		

BALB/c mice, Swiss Albino Mice, Wistar Rats, New Zealand White Rabbits	RCC Laboratories India Private Limited, Hyderabad, India	N/A
**Oligonucleotides**		
RT-PCR Forward Primer: *RdRP*_SARSr-F2-GTGARATGGTCATGTGTGGCGG	Eurofins Analytical Services India Private Limited, Bangalore	N/A
RT-PCR Reverse Primer: *RdRP*_SARSr-R1-CARATGTTAAASACACTATTAGCATA	Eurofins Analytical Services India Private Limited, Bangalore	N/A
P2-FAM CAGGTGGAACCTCATCAGGAGATGC-BHQ1	Eurofins Analytical Services India Private Limited, Bangalore	N/A
Master mix	Thermo Fisher Scientific, USA	catalogue no: EP0441, #R0181
**Software and Algorithms**		
Prism	GraphPad Prism version 8	N/A
R software	R version 4.0.1	N/A
Flow Cytometer	FACS Verse, BD Biosciences, CA, USA	N/A

Experimental Model and Subject Detail

Ethics statements

The studies were conducted post approval from Institutional Animal Ethics Committee (IAEC) and Institutional Biosafety Committee (IBSC) following all ethical practices as laid down in the guidelines for animal care. All studies were performed following both national and international guidelines (CDSCO, 2019).

Experimental animals

Experimental animals *viz*., Wistar rats, Swiss albino mice (SA), BALB/c mice and New Zealand White (NZW) rabbits (*in vivo* models) were sourced from CPCSEA approved vendor. Young adult animals were used and the average body weight ranged from 150 - 200 g in males and 145 - 195 g in females for Wistar rats, 21 - 25 g in males and 17 - 20 g in females for Swiss albino mice, 14 - 16 g in females for BALB/c mice and ~ 2 kg in both males and females for New Zealand White (NZW) rabbits. The studies were conducted in equal number of males and females except in BALB/c mice study where only females were used (**Table 2**).

Animal Husbandry Practices

Maintenance (housing and care) of all animals (mice, rats & rabbits) involved in the study was adhered to guidelines of the Committee for the Purpose of Control and Supervision of Experiments on Animals (CPCSEA, 2003). The animal rooms were air-conditioned with adequate (12 - 15) air changes per hour and provided

with a light cycle of 12 hours light and 12 hours dark. The room temperature was maintained in the range of 22 ± 3°C (rat and mice), 20 ± 3°C (rabbits) and relative humidity between 30 and 70% and it was continuously monitored. Animals were housed in cages with dimensions adhering to CPCSEA guidance. Maintenance Diet and UV Purified water were provided *ad libitum*.

Bacterial Strains:

Salmonella typhimurium strains obtained from Moltox, USA, were used in the Mutagenicity assay, as per the recommendations of OECD guidelines (OECD, 2020)).

Convalescent Serum Samples:

Serum samples were collected from healthy adult volunteers, who were recovered from symptomatic COVID-19 infection i.e., between 21-65 days after virological confirmation. Serum was separated by centrifugation, aliquoted and stored at −80°C until further use. The sera were heat-inactivated at 56°C for 30 min and stored at 4°C prior to analysis.

Cells and Virus

Vero CCL-81 (ATCC# CCL 81) cells were maintained in DMEM supplemented with 10% heat-inactivated fetal bovine serum. Vero cells were revived from GMP master cell bank, which were extensively characterized at BioReliance, USA. SARS-CoV-2 (NIV-2020-770) was obtained from the National Institute of Virology, a WHO Collaborating Center for Emerging Viral Infections(Sarkale et al., 2020), Pune, India and the strain sequence was deposited in the GISAID (EPI_ISL_420545).

Method Details:

1. TCID50

The SARS-CoV-2 virus titer was determined by a cytopathic effect (CPE) method assay. Vero cells ATCC-81 (0.2 x 10^6 cells/mL) were seeded in 96 well plates and incubated for 16- 24 hours at 37 °C. Serial 10-fold dilutions of virus-containing samples were added to 96-well culture plate and cultured for 5-7 days in 5% CO_2 incubator at 37℃, and cells were observed for cytopathic effect (CPE) under a microscope. The virus titer was calculated by the Spearman Karber method (Ramakrishnan, 2016).

2. Virus Inactivation

SARS-CoV-2 Virus (NIV-2020-770) was inactivated with β-propiolactone at a ratio ranging from 1:1500 to 1: 3000 at 2-8 ° C for 24-32 hours and purified by chromatographic purification method. To ensure the effectiveness of the virus inactivation procedure inactivated SARS-CoV-2 virus was inoculated onto veroCCL-

81 monolayers and incubated at 37 °C in a 5% CO_2 incubator and monitored daily for CPE, consecutively for three passages. Further, to reverify the absence of CPE due to supernatant, neat and 10fold dilution of supernatant was inoculated onto Vero cell monolayer and cultured in a 37°C incubator for 5-7 days, and cells were observed for CPE under a microscope.

3. Quantitative Real-Time Reverse Transcription Polymerase Chain Reaction (qRT-PCR)

Total RNA was extracted from the virus sample with a QIAamp Viral RNA mini kit (QIAGEN). SARS-CoV-2 *RdRP-2* gene primer probes sequences are as follows: *RdRP*_SARSr-F2-GTGARATGGTCATGTGTGGCGG,R1-CARATGTTAAASACACTATTAGCATA,P2-FAM CAGGTGGAACCTCATCAGGAGATGC-BHQ1.The SARS-CoV-2 reaction was set up containing a master mix of 10 μL (Thermo) and RNA template 10 μL. qRT-PCR was performed under the following reaction conditions: RT step- 42°C for 30 min for reverse transcription, Initial Denaturation step: 95°C for 3 min and then 45 cycles of Denaturation 95°C for 15 seconds, annealing 58°C for 30 seconds - data acquisition, Extension72°C for 15 seconds. Reactions were set on Biorad-CFX96 as per the manufacturers' instructions.

4. Western blotting

Thirty microliter of samples were mixed with 10 microliters of 4 x sodium dodecyl sulphate polyacrylamide gel electrophoresis (SDS-PAGE) loading buffer, boiled for 10 minutes, and the samples were separated on 10% denaturing SDS polyacrylamide gels by applying constant current. Proteins were transferred from the polyacryamide gel onto a polyvinylidene fluoride (PVDF) membrane for 90 min at 250 mA using semi-dry transblot apparatus. The membrane was blocked with 5% skimmed milk powder (SMP) in PBS overnight at 4°C to reduce non-specific binding. The membrane was incubated with primary antibodies [Rabbit Spike (S1, S2 and RBD) and nucleoprotein antibodies) in 3% SMP (in PBS) at room temperature for 1 h, washed three times with PBS containing 0.05% Tween-20 (PBST), followed by incubation with secondary anti-Rabbit IgG conjugated with horseradish peroxidase in 3% SMP at 37°C for 1 h. The PVDF membranes were then washed three times with PBST, and once with PBS. The luminol-based enhanced chemiluminescence reagent (SignalFire™ ECL Reagent, Cell Signaling Technology, USA) was added to the membrane and X-ray films were exposed to capture the signals obtained on the membrane. The exposed X-ray films were developed and fixed.

5. Experimental Design for both Immunogenicity and safety Evaluation:

Three animal models were used to evaluate both immunogenicity and safety of the three inactivated whole virion adjuvanted vaccine formulations (BBV152 A, B & C) with N+1 dose regimen (1 extra dose than human intended two doses), administered *via* intraperitoneal or intramuscular route. Two animal species (BALB/c

mice & NZW Rabbits) were used to combine both immunogenicity and safety as mentioned under the immunization section. Apart from these studies, repeated dose toxicity studies were also performed with adjuvant alone (Algel and Algel-IMDG) at high dose (30 µg TLR7/8 agonist molecule) in two animal species (Wistar rats and Swiss Albino mice), whereas adjuvanted vaccine formulations containing high dose of antigen (9µg) was tested in Wistar rats. Further, adjuvanted vaccine formulations with actual single human dose (3µg & 6µg) were tested in two animal species (Swiss Albino Mice & Rabbits). All animal studies were conducted with an equal number of adult males and females, unless otherwise stated. Appropriate antigen or adjuvant controls or vehicle control were also maintained, wherever specified.

6. Immunization:

BALB/c Mice: BALB/c mice (inbred mice, 6-8week old) were vaccinated via an intraperitoneal or intramuscular route with either full or 1/10th or 1/20th of human intended single dose of adjuvanted vaccine formulations containing various antigen concentrations (3, 6 & 9 µg). Schematic representation of dosing regimen and dosing schedule is as represented in **Figure 2**.

New Zealand White Rabbits: NZW rabbits (3-4 months old) were vaccinated via an intramuscular route with full Human intended single dose of adjuvanted vaccine formulation/s (BBV152 A, B or C).

Animals were bled from the retro-orbital plexus, and serum was separated and stored at -20°C until further use. Individual sera from all vaccinated species (mice and rabbits) were used to test the antigen-specific antibody binding titer and antibody isotyping profile by Enzyme-Linked Immunosorbent Assay (ELISA) and neutralization antibody titer by Plaque Reduction Neutralization Test ($PRNT_{90}$) or Micro Neutralization Test (MNT_{50}).

7. Enzyme-linked immunosorbent assay (ELISA)

ELISA tests were performed as per standard protocols designed for SARS CoV-2. Microtiter plates were coated with SARS-CoV-2 specific antigens (whole inactivated antigen or spike, S1 /Receptor Binding Domain (RBD)/nucleocapsid (N) at a concentration of 1µg/ml, 100µl/well in PBS pH 7.4). Serially diluted pooled or individual sera from vaccinated animals were added followed by the secondary antibody Goat Anti-mouse IgG HRP (Sigma, USA) conjugated antibody and Goat anti-rabbit IgG HRP conjugate antibody (R&D systems, USA) (dilution 1:2500) for mice and rabbit sera samples respectively. Tetra (3,3′,5,5′) methyl benzidine was used as a substrate. In ELISAs, Threshold (Mean + 3SD) was established by taking the absorbance of negative control (PBS) group, or pre-immune sera and antigen-specific end point titers were determined.

8. Immunoglobulin (IgG) Subclass:

Th1-dependent IgG2a vs. Th2 -dependent IgG1 antibody subclasses were determined by ELISA from mice vaccinated sera as previously described. Briefly, 96 well microtiter plates were coated with spike (S1) protein, at a concentration of 1µg/ml, in PBS pH 7.4) and blocked. Serially diluted individual sera from hyper immunized mice were added followed by the addition of anti-mouse IgG1 or IgG2a or IgG3 HRP conjugate antibodies (dilution 1:2500) After incubation of the plate for 1hr at RT, wells were washed, and 3,3′,5,5′-tetramethylbenzidine (TMB) was added as a substrate to develop color. Threshold (Mean + 3SD) was established by taking the absorbance of negative control (PBS) group, or pre-immune sera and antigen-specific IgG1, IgG2a and IgG3 end point titers were determined.

9. Cytokine (IFNγ & IFNα) Estimation by ELISA:

To determine IFNγ, Enzyme-Linked Immunosorbent Assay (ELISA) was performed according to the instruction manual. Briefly, the capture antibody was coated to plates and blocked. Serial dilutions of Standard or sera samples were prepared and added to wells in triplicates. The plate was further added with detection antibody followed by Avidin-HRP was added and incubated as per described in the manual. Finally, substrate solution was added and 2N $H_2SO_4$was used as stopping solution. The plate was read at 450 nm.

PBMCs cell culture supernatant was used to estimate IFNα using The VeriKine Human Interferon Alpha ELISA Kit (PBL Assay Science, USA). The assay was performed as per the manufacturer’s instructions. Briefly, Pre-coated plates were incubated with diluted standard (range 500-12.5 pg/ml) or culture supernatant, for 1hr at room temperature. Later, the diluted antibody and HRP solution were added sequentially. TMB was used as a substrate, followed by the addition of stop solution. The plate was read at 450nm.

10. Intracellular Staining:

Vaccinated splenocytes (2x10^6/ml) were cultured in 24 well plates and stimulated with inactivated SARS-COV-2 antigen (1.2 µg/ml) or PMA (25 ng/ml, Sigma) and Ionomycin (1 µg/ml, Sigma) along with Protein transport inhibitor (BD biosciences, CA, USA). Cells were washed and centrifuged at 1000rpm for 5-10min and stained with APC-Cy™7 Rat Anti-Mouse CD3 (BD Biosciences, CA, USA), FITC Rat Anti-Mouse CD4 (BD Biosciences), and PE-Cy™7 Rat Anti-Mouse CD8a (BD Biosciences, CA, USA) for 30 minutes at 4°C. Cells were again washed twice with PBS and fixed using fixation/Permeabilize solution (BD Biosciences, CA, USA) for 20 mins at 4°C. Following fixation/permeabilization, cells were washed with 1x permeabilization buffer and stained with intracellular cytokines (IFN-ɣ (BV421 Rat Anti-Mouse IFN-γ, BD Biosciences) for 30 mins at 4°C. Cells were washed and resuspended in 500µl FACS buffer (BD Biosciences). All samples were acquired using BD FACSVerse (BD Biosciences, CA, USA).

11. Cytokine Estimation by Cytokine Bead Array (CBA) assay:

To assess the secretion of multiple Th1 or Th2 mediated cytokines, if any, and to differentiate between Algel1 and Algel2, we used vaccinated mice sera samples collected at various time points (Day 0, 7, 14, 21 & 28, 7 days post-vaccination) and measured Cytokines using the BD CBA Mouse Th1/Th2/Th17 Cytokine Kit (BD Bioscience, San Jose, CA, USA). Sera samples were processed as per the manufacturer's instructions. Samples were measured on the BD FACS Verso and analyzed by FCAP Array Software (BD Bioscience).

12. Plaque Reduction Neutralization Test ($PRNT_{90}$):

The Plaque reduction neutralization test was performed in a biosafety level 3 facility as described earlier (Deshpande et al., 2020). Briefly, Serial dilutions (4 fold) of vaccinated serum samples were mixed with the virus, which can form 50 plaque-forming units and then incubated for 1 h at 37°C. The virus–serum mixtures were added onto the preformed Vero CCL-81 cell monolayers and incubated for 1 h at 37°C in a 5% CO_2 incubator and overlay medium (2% carboxymethyl cellulose with 2% FBS in 2X MEM)was added to cell monolayer, which was further incubated for 4-5days. The number of plaques were counted, and $PRNT_{90}$ were further analyzed using 50% Probit Analysis (Deshpande et al., 2020). A neutralization antibody titer of < 1:20 was considered negative, while that of > 1:20 was considered as positive.

13. Micro Neutralization Test assay (MNT)

The serum of the animal to be tested was inactivated in a 56 ° C -water bath for 30 min. Serum was successively diluted 1:8 to the required concentration by a 2-fold series, and an equal volume of challenge virus solution containing 100 (Cell Culture Infectious Dose 50) $CCID_{50}$viruses was added. After neutralization in a 37°C incubator for two hours, a 1.0 x 10^5 /mL cell suspension was added to the wells (0.1 mL/well) and cultured in a CO_2 incubator at 37℃ for 3-5 days. The Karber method (Ramakrishnan, 2016) by observing the CPE was used to calculate the neutralization endpoint (convert the serum dilution to logarithm), which means that the highest dilution of serum that can protect 50% of cells from infection by challenge with 100 $CCID_{50}$ virus is the antibody potency of the serum. A neutralization antibody potency < 1:20 is negative, while that of > 1:20 is positive.

14. Mutagenicity Assay (Bacterial Reverse Mutation)

The mutagenic potential of the Adjuvant, Algel-IMDG, was evaluated by Bacterial Reverse Mutation assay through plate incorporation and pre-incubation methods using *Salmonella typhimurium* strains TA 1535, TA 1537, TA 98, TA 100, and TA 102 following OECD Guidelines (OECD 2020) either in the presence or absence of S9. Mutagenicitywas assessed either as a reduction in the number of His+ revertants or as an alteration in the auxotrophic background (*i.e*., background lawn).

15. Maximum Tolerated Dose Test or Single Dose Toxicity Study:

Maximum Tolerated Dose (MTD) study was performed in two animal species (Swiss Albino mice and Wistar Rats) species with Algel-IMDG alone with a single maximum dose (20µg TLR7/8 agonist molecule). Animals (Swiss Albino mice and Wistar Rats) were administered via an intramuscular route with Algel-IMDG on day 0 and observed for clinical signs, mortality, and changes in body weight, if any up to 14 days.

16. Repeated dose toxicity:

Repeated dose toxicity studies were performed following both national and international guidelines "Guidelines on the nonclinical evaluation of vaccine adjuvants and adjuvanted vaccines-WHO 2013," n.d.; "OECD Guidelines for Testing of Chemicals, Section 4, No. 471: 'Bacterial Reverse Mutation Test', adopted July 21st, 1997," n.d.; "Schedule Y (Amended version of 2019)of the Drugs and Cosmetics Act 1940 and Rules 1945 of the Government of India," n.d.in compliance with OECD Principles of GLP (). Animals were administered with Adjuvanted vaccine or antigen or adjuvant alone or PBS *via* an intramuscular route with three dose (N+1) regimen with an interval of 7 days (Day 0, 7, and 14).. Recovery groups were maintained, wherever specified, otherwise animals were observed upto 14 days, after the third dose to evaluate recovery effects, if any as a part of safety assessment. All animals were observed for mortality and clinical signs during the experimental period. Animals were bled on Day 2 and also on the day of necropsy (either on day 21, for main groups, or on day 28 for recovery groups) and analyzed for detailed clinical pathology investigations. Organs collected on day 21 (main groups) and/or on day 28 (recovery groups) were evaluated for macroscopic and microscopic findings. See also schematic representation of dosing regimen, dosing schedule and blood collection is as outlined in the **Figure 2**.

Clinical Biochemistry

Blood and urine samples were collected under light isoflurane anesthesia (E-Z anesthesia, Euthanex, USA) and performed clinical evaluations such as hematology (Advia 2120, Siemens), serum chemistry (CobasC111 Analyser, Roche), coagulation parameters (STA Compact®, DiagnosticaStago, France), urinalysis (Urisys® 1800, Roche) and acute phase proteins using the validated ELISA method (Life Diagnostics, USA).

Samples for hematology and clinical biochemistry were collected on day 2 and 21 for main groups and on day 28 for the recovery groups. Urinalysis and Coagulation was performed in rat and rabbit on day 21 and 28. Acute phase protein [Alpha 1-acid glycoprotein (α1-AGP) in rats and mice and C-reactive protein in rabbit] analysis were evaluated in plasma samples collected on days 0 (before dosing), 2 and 21.

Histopathology

Animals were euthanized either on Day 21 (main groups) and/or on day 28 (recovery groups), by carbon dioxide asphyxiation (Smart Box, Euthanex, USA) for rats and mice and using thiopentone for rabbits, and necropsied and observed macroscopically. Organs such as the brain, thymus, spleen, ovaries, uterus, heart, kidneys, testes, liver, adrenals, lungs, epididymides, and prostate with seminal vesicles and coagulating glands were weighed and all organs as per the WHO guidelines were collected for microscopic examinations. Organs for microscopic examination were preserved in 10% neutral buffered formalin (NBF). Tissues were processed (Leica Biosystems, Germany) and stained with hematoxylin and eosin.

17. Statistical Methods

Statistical Analysis was performed in Graph Pad Prism 7.01 by applying two-sided one sample t-test, two sample t-test, MannWhitney and Wilcoxon signed rank test with 5% level of significance for continuous variables

REFERENCES

CPCSEA, 2003. CPCSEA Guidelines for laboratory animal facility. Indian J. Pharmacol. 35, 257.

CDSCO, C., 2019. NewDrugs CTRules 2019.

Deshpande, G.R., Sapkal, G.N., Tilekar, B.N., Yadav, P.D., Gurav, Y., Gaikwad, S., Kaushal, H., Deshpande, K.S., Kaduskar, O., Sarkale, P., Baradkar, S., Suryawanshi, A., Lakra, R., Sugunan, A.P., Balakrishnan, A., Abraham, P., Salve, P., 2020. Neutralizing antibody responses to SARS-CoV-2 in COVID-19 patients. Indian J. Med. Res. 152, 82.

Guidelines on the nonclinical evaluation of vaccine adjuvants and adjuvanted vaccines-WHO 2013, n.d.https://www.who.int/biologicals/areas/vaccines/ADJUVANTS_Post_ECBS_edited_clean_Guidelines_NCE_Adjuvant_Final_17122013_WEB.pdf

OECD, 2020. Test No. 471: Bacterial Reverse Mutation Test, OECD Guidelines for the Testing of Chemicals, Section 4. OECD. https://doi.org/10.1787/9789264071247-en

Sarkale, P., Patil, S., Yadav, P.D., Nyayanit, D.A., Sapkal, G., Baradkar, S., Lakra, R., Shete-Aich, A., Prasad, S., Basu, A., Dar, L., Vipat, V., Giri, S., Potdar, V., Choudhary, M.L., Praharaj, I., Jain, A., Malhotra, B., Gawande, P., Kalele, K., Gupta, N., Cherian, S.S., Abraham, P., 2020. First isolation of SARS-CoV-2 from clinical samples in India. Indian J. Med. Res. 151, 244–250.

Ramakrishnan, M.A., 2016. Determination of 50% endpoint titer using a simple formula. World J. Virol. 5, 85–86.

List of abbreviations and their full form

Abbreviations	Full form
SARS-CoV-2	Severe acute respiratory syndrome coronavirus-2
WHO	World Health Organization
COVID-19	Coronavirus disease-19
SARS	Severe acute respiratory syndrome
MERS	Middle East Respiratory Syndrome
BPL	BPL β-Propiolactone
TCID50	Tissue Culture Infectious Dose 50
NAb	Neutralizing antibody
SD	Standard deviation
CCID50	Cell Culture Infectious Dose 50
MEM	Minimum Essential Media
FBS	Fetal bovine serum
CPCSEA	Committee for the Purpose of Control And Supervision of Experiments on Animals.
PBS	Phosphate-buffered saline
PBS-T	Phosphate-buffered saline with Tween 20
TMB	3',3'5,5'-tetramethylbenzidine
PRNT90	Plaque Reduction Neutralization Test 90
ELISA	Enzyme Linked Immunosorbent Assay
HRP	Horseradish peroxidase
CPE	Cytopathic effect
PCR	Polymerase Chain Reaction
HRP	Horseradish peroxidase
BBIL	Bharat Biotech International Limited
ICMR-NIV	Indian Council of Medical Research-National Institute of Virology
